# Functionalized carbon metal nanocomposites for efficient and sustainable removal of pharmaceutical contaminants from water - A comprehensive review

**DOI:** 10.1016/j.biotno.2026.03.001

**Published:** 2026-04-04

**Authors:** Deepshikaa Kannan, Bellarmin Michael, Elizabethrani Edwin, Jayashree Srinivasan, Sakthi Sanjana, Nandhini Jayaprakash

**Affiliations:** Department of Pharmaceutics, Saveetha College of Pharmacy, Saveetha Institute of Medical and Technical Sciences, Chennai, 602105, India

**Keywords:** Carbon metal nanocomposites, Pharmaceutical contaminants, Water purification, Functionalization strategies, Sustainable remediation, Wastewater treatment

## Abstract

Carbon–metal nanocomposites have emerged as promising materials for the sustainable remediation of pharmaceutical pollutants in aquatic environments by integrating the high surface area, chemical tunability, and structural stability of carbon nanomaterials with the catalytic and redox functionalities of metallic nanoparticles. This review critically evaluates the synthesis strategies, physicochemical characterization, and pollutant removal mechanisms of functionalized carbon–metal nanocomposites, with particular emphasis on adsorption, catalytic degradation, and photocatalytic processes. Carbon-based nanomaterials such as carbon nanotubes, graphene oxide, and carbon dots, when combined with metal nanoparticles including silver, copper, zinc oxide, and iron oxide, exhibit pronounced synergistic effects that enable the efficient and selective removal of a wide range of pharmaceutical contaminants. Environmental and ecotoxicological considerations associated with the application of these nanocomposites are also discussed, highlighting the importance of comprehensive risk assessment and sustainable material design. Despite their significant potential, challenges related to scalability, cost, and regulatory acceptance remain major barriers to practical implementation. Future research directions focus on green synthesis approaches, the development of advanced hybrid nanocomposites, and their integration with complementary water treatment technologies, underscoring the promise of carbon–metal nanocomposites as sustainable solutions for pharmaceutical pollution control and the protection of aquatic ecosystems.

## Graphical abstract

**Figure:**
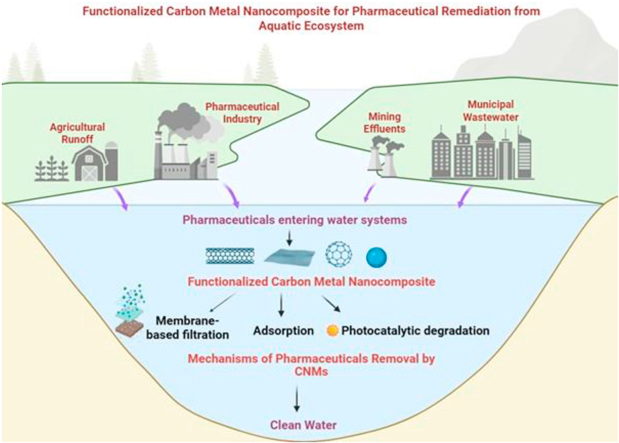

## 1 Introduction

**Fig. 1:**
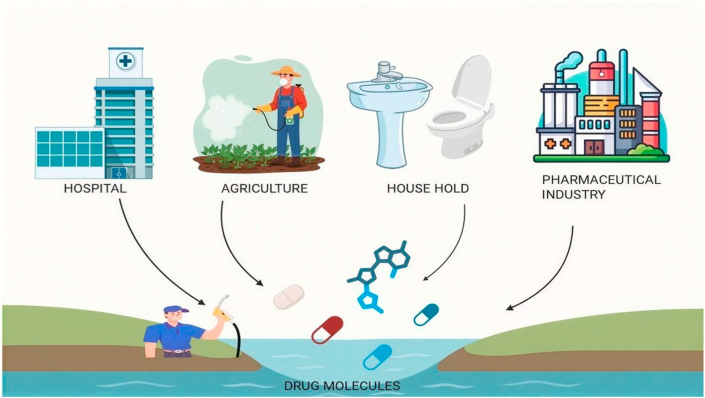
Sources of pharmaceutical contaminations.

Pharmaceutical pollution in aquatic ecosystems has emerged as a critical environmental concern due to the widespread and often unregulated use of pharmaceutical compounds in human healthcare, agriculture, and veterinary practices. Pharmaceuticals, including antibiotics, hormones, analgesics, and endocrine-disrupting compounds, are continuously released into water bodies through multiple pathways such as hospital effluents, pharmaceutical manufacturing discharges, municipal wastewater treatment plant effluents, and agricultural runoff, leading to their persistent presence in aquatic environments.^1^^,^^2^ Fig. 1 illustrates the major sources of pharmaceutical contamination in aquatic environments. Unlike conventional pollutants, pharmaceutical residues are biologically active, persistent, and capable of eliciting adverse ecological and toxicological effects even at trace concentrations.

Pharmaceutical compounds are now recognized as ubiquitous contaminants in wastewater systems worldwide. Monitoring studies consistently report the occurrence of multiple pharmaceutical classes in municipal and hospital wastewater, with detection frequencies often exceeding 70–90% of analyzed samples. Concentrations in raw wastewater typically range from nanograms per liter (ng L^−1^) to several micrograms per liter (μg L^−1^), depending on compound properties, consumption patterns, and the efficiency of wastewater treatment infrastructure. Antibiotics, analgesics, and anti-inflammatory drugs are among the most frequently detected compounds, commonly reported at concentrations between 0.1 and 50 μg L^−1^ in influent wastewater, while treated effluents often still contain pharmaceutical residues at levels ranging from tens to hundreds of ng L^−1^.^3^, ^4^

In municipal wastewater, hospital effluents, and receiving surface waters, the most frequently reported pharmaceutical contaminants include nonsteroidal anti-inflammatory drugs (NSAIDs) such as diclofenac, ibuprofen, and naproxen; the antiepileptic drug carbamazepine; antibiotics including sulfamethoxazole, ciprofloxacin, and trimethoprim; and lipid-regulating agents such as gemfibrozil and bezafibrate.^5^ Occurrence studies conducted in wastewater treatment plants and receiving rivers commonly report these pharmaceutical compounds at concentrations ranging from low ng L^−1^ to several μg L^−1^ in influent wastewater and surface waters, reflecting their high consumption rates, environmental persistence, and incomplete removal by conventional wastewater treatment processes.^6^ Hormonal compounds, including 17α-ethinylestradiol and natural estrogens such as estrone and 17β-estradiol, are also recurrently detected at ng L^−1^ levels, at which they can induce endocrine-disrupting effects in aquatic organisms even at very low concentrations.^7^^,^^8^ These occurrence patterns indicate that a limited group of high-use and environmentally persistent pharmaceuticals dominates the contaminant burden in aquatic systems and therefore represents a primary target for advanced remediation strategies. Conventional wastewater treatment plants typically achieve removal efficiencies of only 20–70% for many pharmaceutical compounds, leading to their continuous release into surface waters and groundwater. The widespread occurrence of these contaminants, coupled with their incomplete removal, underscores the urgent need for advanced, selective treatment technologies capable of effectively addressing pharmaceutical pollutants at environmentally relevant concentrations.^9^

Regulatory frameworks governing pharmaceuticals in aquatic environments remain limited and fragmented, with only a small subset of compounds subject to explicit environmental or drinking-water standards in most jurisdictions. In the European Union, pharmaceuticals are primarily addressed through the Water Framework Directive Watch List mechanism and proposed environmental quality standards for selected high-priority substances, whereas in the United States many pharmaceuticals are included in successive Contaminant Candidate Lists rather than being regulated through enforceable Maximum Contaminant Levels. The World Health Organization considers that pharmaceuticals typically occur in drinking water at trace concentrations that pose a low direct risk to human health and therefore advocates a risk-based water safety plan approach rather than establishing individual guideline values for most pharmaceutical compounds. In India, regulatory efforts have primarily focused on controlling emissions from pharmaceutical manufacturing and addressing antimicrobial resistance, with the Central Pollution Control Board issuing guidelines for monitoring active pharmaceutical ingredient residues in industrial effluents, along with draft standards proposing stringent discharge limits for antibiotics and bulk drug manufacturing facilities.^10^

**Fig. 2:**
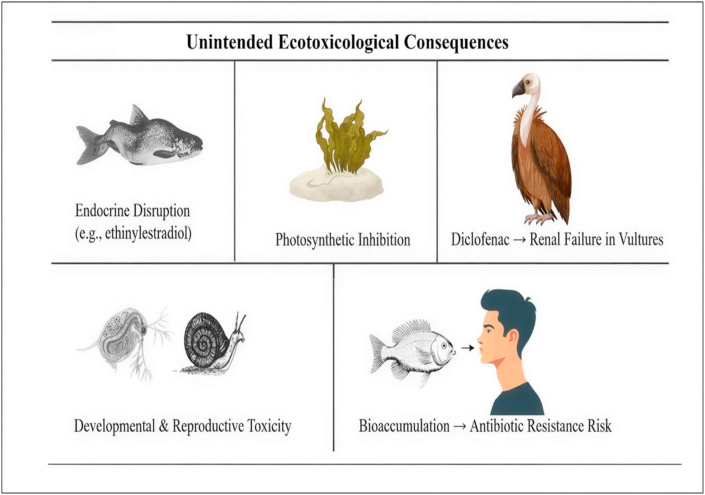
Unintended ecotoxicological consequences of pharmaceutical.

The environmental impact of pharmaceutical contaminants extends beyond direct ecological toxicity and encompasses significant public health concerns. Many pharmaceutical compounds interfere with the physiological, developmental, and reproductive processes of aquatic organisms, leading to adverse effects such as endocrine disruption, growth inhibition, and altered reproductive behaviour. Fig. 2 illustrates the unintended ecotoxicological consequences associated with pharmaceutical and personal care product contamination, including endocrine disruption in fish, photosynthetic inhibition in algae, renal failure in vultures resulting from diclofenac exposure, developmental and reproductive toxicity in aquatic invertebrates, and bioaccumulation across aquatic food webs.

Furthermore, the presence of antibiotics in aquatic environments accelerates the emergence and dissemination of antibiotic-resistant microorganisms, posing a significant global public health threat.^11^ It is also important to note that pharmaceutical residues in wastewater are typically present at ng L^−1^ to sub-ng L^−1^ concentrations and therefore require highly sensitive analytical workflows, most commonly solid-phase extraction coupled with liquid chromatography–tandem mass spectrometry (LC–MS/MS), while simpler high-performance liquid chromatography–UV methods or immunoassays are generally limited to preliminary screening or applications involving less complex matrices.^12^ Pharmaceutical pollutants have traditionally been treated using conventional wastewater treatment processes such as coagulation, sand filtration, activated carbon adsorption, ozonation, and chlorination. Although these methods are effective for the removal of bulk organic matter and pathogenic microorganisms, they often exhibit limited efficiency toward pharmaceutical compounds due to their high polarity, chemical stability, and resistance to biodegradation under conventional treatment conditions.^13^

Activated carbon, although widely employed, often lacks selectivity and is associated with high regeneration and operational costs, while chemical oxidation processes may lead to the formation of toxic transformation by-products. These limitations underscore the urgent need for more efficient, selective, and sustainable remediation strategies. Among emerging treatment approaches, adsorption-based technologies have attracted considerable attention owing to their operational simplicity, high effectiveness, and flexibility in water treatment applications.^14^

Carbon-based adsorbents, including activated carbon, biochar, carbon nanotubes, graphene oxide, and carbon dots, have been extensively investigated for the removal of pharmaceutical pollutants due to their high surface area, tunable pore structure, and strong physicochemical interactions with organic molecules. Nanostructured carbon materials generally exhibit higher adsorption capacities and faster adsorption kinetics than conventional adsorbents, making them promising candidates for advanced and efficient water treatment applications.^15^ However, pristine carbon nanomaterials often exhibit several limitations, including aggregation in aqueous environments, limited selectivity toward specific pharmaceutical compounds, diminished performance in the presence of natural organic matter, and challenges related to regeneration and reuse. To address these drawbacks, nanotechnology-driven surface functionalization strategies have been extensively investigated. Such functionalization enhances the physicochemical properties of carbon nanomaterials by introducing active functional groups or catalytic components, thereby improving dispersibility, adsorption efficiency, and pollutant degradation performance.

In recent years, the integration of carbon nanomaterials with metal and metal oxide nanoparticles, such as silver, copper, zinc oxide, and iron-based materials, has emerged as a highly effective strategy for the remediation of pharmaceutical pollutants in aquatic environments.^16^ These functionalized carbon–metal nanocomposites integrate the high adsorption capacity of carbon materials with the catalytic, redox, or photocatalytic activities of metallic components, enabling the simultaneous adsorption and degradation of pharmaceutical pollutants. Such synergistic systems exhibit enhanced removal efficiency, improved selectivity, and greater reusability compared with single-component materials.^17^

Despite significant progress, a comprehensive understanding of the performance of functionalized carbon–metal nanocomposites under environmentally relevant conditions remains limited. Many existing studies rely on idealized laboratory settings and do not sufficiently address critical challenges such as interference from natural organic matter, long-term stability, scalability, and environmental safety. Accordingly, the present review provides an integrated and critical evaluation of recent advances in functionalized carbon–metal nanocomposites for the remediation of pharmaceutical pollutants.^18^ By systematically linking synthesis strategies, structure–property relationships, pollutant removal mechanisms, environmental interactions, and practical limitations, this review identifies key research gaps and outlines future directions aimed at bridging laboratory-scale research with real-world water treatment applications. Through a structured past–present–future perspective, functionalized carbon–metal nanocomposites are positioned as promising materials for the development of sustainable water purification technologies.

## 2 Review methodology

A systematic and comprehensive literature search was conducted to identify studies focusing on functionalized carbon–metal nanocomposites for the remediation of pharmaceutical pollutants in aquatic environments. Major scientific databases, including Scopus, Web of Science, PubMed, Google Scholar, and ScienceDirect, were systematically searched to ensure broad coverage of peer-reviewed literature. The search period primarily spanned January 2020 to December 2025, allowing the inclusion of both recent advancements and key foundational studies relevant to emerging research trends. A structured combination of targeted keywords was employed to capture diverse aspects of carbon-based nanocomposites and their remediation mechanisms. The primary search terms included carbon nanomaterials, functionalized carbon, carbon–metal nanocomposites, graphene oxide, carbon nanotubes, carbon dots, metal nanoparticles, pharmaceutical contaminants, water purification, photocatalytic degradation, adsorption, and advanced oxidation processes. Boolean operators and keyword combinations were applied to refine the search strategy and enhance the relevance of retrieved records.

Studies were selected based on predefined inclusion and exclusion criteria to ensure scientific rigor and relevance. Inclusion criteria comprised peer-reviewed research articles and review papers investigating carbon-based nanomaterials functionalized with metals or metal oxides for pharmaceutical pollutant removal. Eligible studies addressed adsorption, photocatalysis, catalytic degradation, or hybrid remediation approaches and reported clear experimental methodologies along with quantifiable performance metrics, such as removal efficiency, degradation rate, or adsorption capacity. Studies were excluded if they were not directly related to water purification or pharmaceutical contaminant remediation. In addition, articles focusing solely on standalone metal nanoparticles without a carbon-based component, as well as studies lacking sufficient methodological detail or measurable performance outcomes, were excluded. This systematic screening process ensured that the reviewed literature was methodologically robust and closely aligned with the objectives of the present review.

## 3 Fundamentals of carbon nanomaterials

### 3.1 Types of carbon nanomaterials

Among carbon-based nanomaterials, carbon nanotubes (CNTs), graphene oxide (GO), and carbon dots (CDs) are among the most extensively investigated due to their distinctive physicochemical properties. CNTs, consisting of rolled graphene sheets arranged in a cylindrical structure, exhibit exceptional tensile strength, high thermal stability, and large specific surface area, rendering them highly effective candidates for the adsorption and catalytic degradation of environmental pollutants.^19^ Graphene oxide (GO), in contrast, consists of a single-atom-thick layer of sp^2^-hybridized carbon atoms decorated with oxygen-containing functional groups, including hydroxyl, epoxy, and carboxyl moieties. These functional groups significantly enhance its hydrophilicity and dispersibility in aqueous media, thereby promoting strong interactions with polar pharmaceutical contaminants.^20^

The adsorption performance of carbon-based nanomaterials can be substantially enhanced through surface functionalization and hybridization strategies. For example, functionalization with poly (amidoamine) (PAMAM) dendrimers has been shown to significantly improve the adsorption capacity of graphene and carbon nanotubes by providing a high density of accessible binding sites. Molecular dynamics simulations, supported by experimental validation, have demonstrated that PAMAM-functionalized graphene and carbon nanotubes exhibit markedly enhanced affinity toward heavy metal ions such as uranyl, particularly when higher-generation dendrimers are employed. This enhancement is primarily attributed to the increased number of terminal amine groups available for metal coordination, which strengthens intermolecular interactions and leads to improved adsorption efficiency.^21^

Such modifications not only broaden the spectrum of target contaminants but also enable precise tailoring of surface chemistry to meet specific application requirements. Collectively, the distinctive structural features and surface functionalities of carbon nanotubes, graphene oxide, and carbon dots render them highly effective for the removal of a wide range of pharmaceutical pollutants. Moreover, their inherent adaptability allows these materials to be employed either as standalone adsorbents or as integral components of multifunctional hybrid systems, thereby further enhancing their potential for advanced water treatment technologies.^22^

### 3.2 Physical and chemical properties of carbon nanomaterials

The physicochemical characteristics of carbon nanomaterials play a critical role in determining their effectiveness in pharmaceutical pollutant remediation. Carbon nanotubes, characterized by their high aspect ratio and large specific surface area, facilitate the adsorption of a wide range of pharmaceutical compounds through mechanisms such as π–π stacking, hydrophobic interactions, and van der Waals forces.^23^ Graphene oxide (GO), with its unique two-dimensional lamellar architecture and abundant oxygen-containing functional groups—including hydroxyl, carboxyl, and epoxy moieties—exhibits excellent dispersibility in aqueous media and provides a high density of active sites suitable for both direct adsorption and subsequent chemical modification.^24^

In contrast, carbon dots (CDs) offer distinct advantages arising from their nanoscale dimensions, high surface-to-volume ratio, and diverse surface chemistry. Functional groups such as amines and hydroxyls not only enhance their aqueous solubility but also facilitate strong interactions with polar pharmaceutical molecules. Moreover, CDs exhibit unique photophysical properties, including tunable fluorescence and the ability to function as both electron donors and acceptors, making them promising candidates for redox-driven degradation processes and as photosensitizers in visible-light-assisted photocatalytic systems.^25^

The integration of carbon dots (CDs) with other carbon nanomaterials, such as graphene oxide (GO), further enhances their remediation performance. For example, GO–CD nanocomposites have demonstrated pronounced synergistic effects in the adsorption and visible-light-driven photocatalytic degradation of pharmaceutical contaminants such as tetracycline. The presence of nitrogen- and oxygen-containing functional groups on both components improves dispersibility in aqueous media and provides a high density of reactive sites. In addition, the efficient electron transfer capability of CDs complements the large surface area of GO, facilitating enhanced charge separation and the generation of reactive species required for redox reactions and effective contaminant degradation.^26^ Taken together, these carbon nanomaterials constitute a versatile and complementary toolkit for environmental remediation applications. By precisely tuning their surface functionalities, structural configurations, and composite architectures, carbon nanomaterials can be strategically engineered to optimize the adsorption, removal, and degradation of pharmaceutical residues from aquatic systems.^18^

## 4 Metallic nanoparticles and functionalization strategies for enhanced carbon-based remediation

### 4.1 Common metal nanoparticles used in remediation

Metal nanoparticles (MNPs) have attracted considerable attention as effective agents for environmental remediation, particularly for the degradation of persistent pharmaceutical pollutants in aquatic systems. Their nanoscale dimensions confer a high surface-area-to-volume ratio, unique optical and electronic properties, and strong redox activity, making them well suited for both adsorption and catalytic degradation processes. Commonly investigated MNPs include silver, copper, zinc, and iron-based nanoparticles, each of which exhibits distinct physicochemical properties and pollutant removal mechanisms, such as redox reactions, reactive oxygen species generation, and surface-mediated catalytic transformations.^27^

Silver nanoparticles are particularly notable for their strong antimicrobial activity. Through the controlled release of Ag^+^ ions, they disrupt microbial respiratory enzymes, induce oxidative stress, and compromise cellular membrane integrity, rendering them especially valuable in wastewater treatment applications aimed at controlling antibiotic-resistant bacteria.^28^ When integrated with magnetic cores, such as in Fe_3_O_4_–Ag nanoparticle nanocomposites, silver-based systems offer the combined advantages of high adsorption capacity and facile magnetic recovery. These core–shell architectures have demonstrated rapid and efficient removal of pharmaceutical contaminants such as ibuprofen, achieving adsorption efficiencies exceeding 90% within 45 min under neutral conditions. The adsorption behavior is commonly well described by the Langmuir isotherm model, indicating monolayer adsorption on homogeneous active sites. Moreover, these nanocomposites exhibit excellent reusability, retaining more than 89% of their initial adsorption efficiency after three regeneration cycles. Comprehensive physicochemical characterization using techniques such as scanning electron microscopy (SEM), energy-dispersive X-ray spectroscopy (EDX), Fourier-transform infrared spectroscopy (FTIR), Brunauer–Emmett–Teller (BET) surface area analysis, and differential scanning calorimetry (DSC) further confirms the structural stability, surface functionality, and recyclability of these materials.^29^

In addition to silver nanoparticles, copper and zinc nanoparticles provide complementary advantages in pharmaceutical pollutant remediation. Copper- and zinc-based nanoparticles exhibit notable antimicrobial activity and redox reactivity, which can be effectively harnessed for the degradation of complex pharmaceutical molecules.^30^ Zinc oxide (ZnO) nanoparticles, for example, are widely utilized for their strong photocatalytic activity. Upon activation under ultraviolet or visible light irradiation, ZnO generates reactive oxygen species (ROS), including hydroxyl and superoxide radicals, which effectively attack and degrade a broad range of pharmaceutical compounds.^31^

Iron-based nanoparticles, including zero-valent iron (ZVI), Fe_2_O_3_, and Fe_3_O_4_, play a critical role in advanced oxidation processes for pharmaceutical pollutant remediation. These materials function as effective catalysts in Fenton and photo-Fenton reactions, promoting the generation of highly reactive hydroxyl radicals (•OH) capable of non-selectively degrading pharmaceutical contaminants, often leading to near-complete mineralization. Recent studies have demonstrated that hybrid systems combining ZnO and Fe_3_O_4_ nanoparticles exhibit enhanced degradation performance, wherein reactive oxygen species generated by ZnO synergistically interact with hydroxyl radicals produced through Fe_3_O_4_-mediated reactions. Under visible-light irradiation, such coupled systems have achieved removal efficiencies of up to 95% for pharmaceutical compounds such as sulfamethoxazole within relatively short reaction times.^32^

By integrating metal nanoparticles into remediation platforms, highly reactive and multifunctional systems can be developed that are capable of addressing a broad spectrum of pharmaceutical pollutants with diverse chemical structures. Their dual functionality as both adsorbents and catalysts, together with the potential for magnetic recovery and photocatalytic regeneration, positions metal nanoparticles as critical components in the advancement of next-generation water treatment technologies.^33^

### 4.2 Functionalization of carbon nanomaterials with organic and inorganic components

Carbon nanomaterials, including carbon nanotubes, graphene oxide, and carbon dots, have attracted increasing attention in environmental remediation owing to their large specific surface area, distinctive morphological features, and highly tunable surface chemistry.^34^ To further enhance their pollutant removal performance, carbon nanomaterials are frequently functionalized using organic or inorganic modification strategies. Organic functionalization commonly involves the introduction of carboxyl (–COOH), hydroxyl (–OH), amine (–NH_2_), or sulfonic acid (–SO_3_H) groups onto the carbon surface through methods such as acid oxidation, covalent grafting, or polymer modification. These surface functionalities markedly improve aqueous dispersibility, strengthen interactions with pharmaceutical contaminants through electrostatic attraction and hydrogen bonding, and provide reactive sites for subsequent conjugation or immobilization, thereby enhancing overall remediation efficiency.^35^

The influence of surface forces on adsorption efficiency has also been quantitatively demonstrated in vertically aligned carbon nanotube (CNT) membranes during the removal of steroid hormones. For example, ethinylestradiol adsorption reached 0.40 ± 0.03 ng cm^−2^ under conditions of low hydrodynamic drag but decreased sharply with increasing flow-induced shear stress. A near-perfect correlation (R^2^ = 0.98) between van der Waals interaction energy and hormone adsorption under intermediate drag conditions highlights the critical role of nanoscale interaction forces in governing adsorption behavior in CNT-based systems. These findings emphasize the importance of tailoring surface chemistry and interfacial properties to modulate molecular-scale forces and achieve efficient pollutant capture.^36^

In contrast, inorganic functionalization involves the incorporation of metal or metal oxide nanoparticles, such as silver, zinc oxide, titanium dioxide, or magnetite, onto carbon nanostructures. This approach results in hybrid carbon–metal nanocomposites that exhibit pronounced synergistic effects, wherein the carbon component provides a high surface area and π-electron-rich domains for effective adsorption, while the inorganic component contributes catalytic or photocatalytic pathways for the degradation of pharmaceutical pollutants.^37^ For example, graphene oxide decorated with silver nanoparticles enables both the adsorption of antibiotics such as ciprofloxacin and their subsequent oxidative degradation through silver-mediated redox activity. Similarly, doping carbon dots with zinc or iron imparts visible-light-responsive photocatalytic properties, facilitating the simultaneous adsorption and degradation of pharmaceutical pollutants. These hybrid nanostructures also demonstrate enhanced colloidal stability, reduced nanoparticle aggregation, and improved accessibility of active sites, thereby increasing their effectiveness in aqueous remediation environments.^38^

By employing tailored functionalization strategies—either organic or inorganic—carbon nanomaterials can be transformed into highly selective and efficient platforms for the removal and degradation of emerging contaminants from water. The combined influence of surface chemistry, interfacial forces, and synergistic catalytic functions underscores the significant potential of engineered carbon nanomaterials in advanced water treatment technologies.^39^

### 4.3 Tuning functional groups for selective pharmaceutical pollutant removal

The functionalization of carbon nanomaterials with metal and metal oxide nanoparticles has emerged as an effective strategy for enhancing both the selectivity and reactivity of these materials toward pharmaceutical contaminants in aqueous environments.^40^ This approach extends beyond conventional surface modification by introducing catalytic and electron-conductive sites that interact selectively with pharmaceutical molecules, thereby enabling simultaneous adsorption and degradation. Metals such as silver, copper, and iron, along with metal oxides including zinc oxide, iron oxide, and cerium oxide, are commonly anchored onto carbon nanomaterials such as carbon nanotubes, graphene oxide, and carbon dots, resulting in multifunctional nanocomposites with optimized and tunable pollutant removal performance.^41^

For example, the integration of Fe_3_O_4_ and TiO_2_ nanoparticles onto graphene oxide (GO) platforms introduces redox-active sites and enhances electronic conductivity, collectively facilitating the degradation of persistent pharmaceutical compounds through surface complexation, interfacial charge transfer, and catalytic oxidation pathways. GO–Fe_3_O_4_ nanocomposites, in particular, have demonstrated enhanced binding affinity toward negatively charged antibiotics due to stronger electrostatic interactions, while the magnetic properties of Fe_3_O_4_ additionally enable facile post-treatment recovery and reuse.^42^ Similarly, carbon scaffolds decorated with ZnO and TiO_2_ nanoparticles exhibit enhanced photocatalytic performance under solar or artificial light, leading to increased generation of reactive oxygen species (ROS) such as hydroxyl radicals (•OH) and superoxide anions (O_2_•^-^). These ROS play a pivotal role in the oxidative degradation of complex pharmaceutical compounds, including sulfonamides and β-lactam antibiotics.^43^

Nanocomposites integrating NiFe_2_O_4_ and CeO_2_ nanoparticles onto graphene oxide have demonstrated exceptional photocatalytic performance, achieving over 95% degradation of tetracycline under visible-light irradiation within 90 min. Mechanistic studies, including molecular docking and density functional theory (DFT) calculations, confirmed the pivotal role of reactive oxygen species (ROS) in driving the degradation process. Additionally, these nanocomposites exhibit antibacterial activity against *Escherichia coli* and *Staphylococcus aureus*, highlighting their potential for dual-function wastewater treatment applications that combine pharmaceutical pollutant removal with microbial disinfection.^44^

Carbon dots, owing to their ultra-small size and abundant edge functional groups, provide an excellent platform for integration with metal oxides. Doping carbon dots with nanoparticles such as MnO_2_ or CuO enhances their oxidative potential and promotes selective recognition of antibiotic molecules. This dual functionality enables these hybrid nanomaterials to serve simultaneously as adsorbents and photocatalysts. Furthermore, controlled doping with transition metals allows fine-tuning of electronic bandgaps and surface energies, optimizing interaction dynamics with specific pharmaceutical targets.^45^ For instance, MnO_2_-doped carbon dots synthesized via green methods using eucalyptus extract have demonstrated rapid degradation of dyes such as methylene blue under visible-light irradiation, achieving over 95% removal within 90 min. These nanomaterials also exhibit excellent reusability, retaining more than 90% of their performance over multiple cycles.

Overall, the synergy between carbon nanostructures and metal-based functionalities enhances contaminant specificity, broadens the spectrum of activity, and enables the development of recyclable, high-performance materials. Such hybridization strategies not only improve the removal efficiency of antibiotics and other pharmaceutical pollutants but also support the long-term sustainability of advanced water treatment technologies. Table 1 summarizes representative functionalized carbon–metal nanocomposites employed for pharmaceutical removal, detailing their target compounds, relative photocatalytic efficiencies, advantages, and limitations.

**Table 1:** Representative functionalized carbon metal nanocomposites for pharmaceutical removal and their key performance characteristics.

Metal type	Pharmaceutical product	Removal efficiency	Advantages	limitations
TiO_2_-rGO	Ibuprofen, sulfamethoxazole, carbamazepine	≈90–100% degradation within 1–2 h under UV/visible light	Strong adsorption + enhanced charge separation, good visible-light response, TiO_2_ is chemically stable.	Gradual oxidation/aging of rGO over multiple cycles, need to prevent particle release, limited real-effluent data.^12^
GO–TiO_2_/GO–ZnO/GO–Ag	Diclofenac, ibuprofen, ciprofloxacin, tetracycline	Typically >80–95% removal under UV/visible light with faster kinetics than unsupported oxides.	Synergistic adsorption photocatalysis, broad-spectrum activity, GO improves dispersibility and electron transport.	ZnO photocorrosion and Zn^2+^ leaching; Ag^+^/AgNP release risk, multi step synthesis, long term detachment of nanoparticles must be controlled.^12^
Fe_3_O_4_@SiO_2_@BiFeO_3_/rGO and other Fe-based CNT/GO composites	Ciprofloxacin, tetracycline, ketoprofen, naproxen	95–98% degradation of fluoroquinolones/tetracyclines under visible light; >90% removal of NSAIDs in Fenton/photo-Fenton or CWPO systems.	Magnetic separation and reuse, combined adsorption and (photo)Fenton oxidation, good performance in complex matrices.	Fe leaching and iron-sludge generation, activity loss after many cycles, pH-sensitive processes, CNT/GO nanosafety issues if not fully retained.^46^^,^^47^
CNT–Ag/CNT–ZnO	Sulfamethoxazole, tetracycline, mixed antibiotics	near-complete removal of several antibiotics in ≤1 h frequently reported.	Very strong adsorption–photodegradation, simultaneous antimicrobial action, effective at low doses.	High material cost, Ag^+^/Zn^2+^ leaching and ecotoxicity concerns, tighter regulatory scrutiny, CNT aggregation/fouling.^48^
CD–metal oxides (CDs/BiOCOOH/g-C_3_N_4_, CDs–MnO_2_)	Sulfathiazole, tetracycline, other sulfonamides	>90–95% degradation within 60–90 min under visible LED/solar-simulated light.	Excellent visible-light harvesting and charge separation, tunable surface chemistry often prepared via green, low-temperature routes.	Limited long-term stability and scale-up data, CD photostability and metal leaching in real wastewater still unclear.^12^
Biochar/biopolymer metal carbon (CuO/Cu_2_O/biochar, magnetic guar-gum/GO)	Carbamazepine, diclofenac, ciprofloxacin, paracetamol	Moderate to high, typically 70–95% removal depending on light, oxidant, and contact time, often dominated by adsorption.	Low-cost, renewable carbon sources, good adsorption capacities, magnetic or Cu-assisted regeneration possible, higher sustainability potential.	Batch-to-batch variability, incomplete mineralization, Cu or other metals can leach in acidic/saline media, less uniform structure than engineered CNT/GO.^49^

## 5 Carbon metal nanocomposites: synthesis and characterization

The development of carbon–metal nanocomposites has gained considerable attention as a versatile platform for the sustainable remediation of pharmaceutical pollutants. The integration of carbon nanomaterials with metallic or metal oxide nanoparticles imparts synergistic properties, including high surface area, chemical tunability, and catalytic reactivity, which are critical for the efficient adsorption and degradation of pharmaceutical residues in aquatic environments. The performance of these hybrid nanostructures is strongly influenced by their synthesis methods and physicochemical characteristics, which govern their structural integrity, stability, and functional reactivity.^50^

### 5.1 Synthesis Methods of Carbon metal nanocomposites

A variety of synthetic strategies have been developed to fabricate carbon–metal nanocomposites, with careful control over nanoparticle dispersion, size, and interfacial compatibility. Chemical vapor deposition (CVD) is commonly employed to grow carbon nanotubes or graphene-based structures in the presence of metal catalysts, promoting the formation of strong metal–carbon interfaces. Sol–gel methods, on the other hand, enable uniform deposition of metal oxides such as ZnO, TiO_2_, and Fe_2_O_3_ onto carbon substrates through the hydrolysis and polycondensation of metal precursors under controlled pH and temperature conditions.^51^

Hydrothermal and solvothermal synthesis methods are particularly advantageous for producing carbon dot–metal oxide hybrids or graphene-supported metal nanoparticles, as they offer improved crystallinity and precise particle size control under moderate temperatures and pressures. Recent advancements include a one-pot sol–gel approach for synthesizing Fe_3_C/few-layered graphene core–shell nanoparticles embedded within a carbon matrix. In this method, oleic acid and oleylamine act as soft surfactants to direct the formation of uniform nanostructures, resulting in materials with a saturation magnetization of approximately 43 emu, which is beneficial for magnetic separation and targeted environmental remediation applications.^52^

**Fig. 3:**
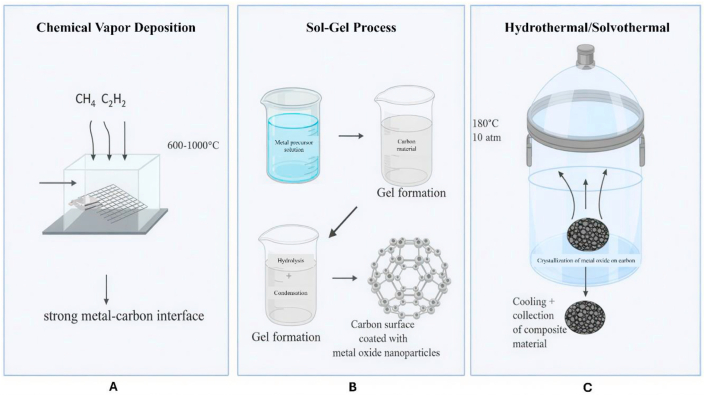
Synthesis methods of carbon–metal nanocomposites.

These synthetic strategies are not only effective for tuning the structural and functional properties of carbon–metal hybrids but are also valued for their scalability and energy efficiency. Furthermore, the adoption of green synthesis approaches using plant extracts, biogenic reducing agents, or environmentally benign solvents enhances their potential for sustainable nanomaterial development in environmental remediation. Fig. 3 provides a schematic overview of commonly employed synthesis methods for carbon-based metal oxide nanocomposites:

(A) **Chemical Vapor Deposition (CVD):** Facilitates the formation of strong metal–carbon interfaces at high temperatures (600–1000 °C) using hydrocarbon precursors.
(B) **Sol–Gel Process:** Involves hydrolysis and condensation of metal precursor solutions to form a gel, followed by deposition of metal oxide nanoparticles onto the carbon substrate.
(C) **Hydrothermal/Solvothermal Synthesis:** Promotes the crystallization of metal oxides on carbon materials under elevated temperature and pressure, followed by cooling and recovery of the composite material.

### 5.2 Characterization techniques

A comprehensive understanding of the morphology, composition, and bonding environment of carbon–metal nanocomposites is essential for optimizing their performance in environmental applications. Advanced characterization techniques are pivotal in this context. Scanning electron microscopy (SEM) and transmission electron microscopy (TEM) offer detailed insights into surface morphology, nanoparticle dispersion, and structural uniformity of the composites.^53^ Transmission electron microscopy (TEM) provides atomic-scale resolution, enabling visualization of core–shell architectures and the distribution of embedded metal or metal oxide nanoparticles within carbon matrices. Complementing these imaging techniques, X-ray photoelectron spectroscopy (XPS) is routinely used to determine elemental composition and oxidation states, thereby confirming the successful incorporation of metal species and their chemical interactions with the carbon framework.^54^

Fourier-transform infrared spectroscopy (FTIR) is instrumental in identifying surface functional groups and monitoring their involvement in pollutant adsorption or post-synthesis modifications. Simultaneously, X-ray diffraction (XRD) provides critical information on the crystallinity and phase composition of both the carbon and metal components. For instance, the synthesis of vanadium nitride (VN)/carbon nanocomposites via thermal decomposition of vanadyl phthalocyanine (VOPC) demonstrated the successful integration of VN nanoparticles within an amorphous carbon matrix.^55^ Comprehensive characterization using SEM, TEM, EDS, FTIR, XRD, and XPS confirmed the presence of a cubic VN phase (space group Fm-3m) and V(III)–N bonding states, validating the structural integrity and chemical stability of the nanocomposite. These observations were further corroborated by electrochemical analyses, which revealed high specific capacity and near-ideal Coulombic efficiency over repeated cycles, highlighting the functional robustness of the synthesized material. Collectively, these characterization techniques not only confirm structural and chemical configurations but also provide critical guidance for optimizing synthesis parameters to enhance performance, durability, and application-specific functionality, particularly in wastewater treatment, photocatalysis, and advanced oxidation processes.^56^

### 5.3 Morphology, surface area, and structural properties

The environmental performance of carbon–metal nanocomposites is closely associated with their morphological and textural properties. A high specific surface area, typically measured using Brunauer–Emmett–Teller (BET) analysis, enhances adsorption capacity and promotes more extensive interactions between pollutants and reactive sites. Additionally, porosity—particularly a balanced combination of microporous and mesoporous structures—facilitates efficient diffusion and entrapment of pharmaceutical contaminants within the composite matrix. Morphological features, including tubular frameworks, sheet-like surfaces, and spherical nanodots, significantly influence water flow dynamics and pollutant accessibility, thereby governing the kinetics of adsorption and catalytic degradation. Equally critical is the uniform distribution of metal nanoparticles on the carbon substrate, which maximizes the availability of catalytic sites and ensures consistent reactivity. Strong interfacial compatibility between the metal and carbon components further enhances structural stability, minimizes the risk of nanoparticle leaching, and improves long-term durability under aqueous conditions.^57^

For example, magnetic biochar-supported NiFe_2_O_4_ composites have been designed with high BET surface areas exceeding 300 m^2^/g and hierarchical pore architectures. This structural optimization markedly enhances the removal efficiency of antibiotics such as ciprofloxacin, achieving adsorption capacities approaching 69 mg/g. High-resolution imaging confirmed the uniform dispersion of NiFe_2_O_4_ nanoparticles on biochar surfaces, ensuring optimal contact with target pollutants. The adsorption process follows pseudo-second-order kinetics and Langmuir isotherm behavior, indicating monolayer chemisorption. Thermodynamic analyses reveal that the process is endothermic and spontaneous, while mechanistic investigations highlight the key roles of π–π interactions, coordination bonding, and hydrogen bonding in pollutant capture.^58^

The composite maintained high removal efficiency over multiple reuse cycles, demonstrating structural resilience and practical applicability. Beyond surface area and morphology, intrinsic defects—such as oxygen vacancies or heteroatom doping within the carbon framework—play a critical role in modulating electron transfer and catalytic activity. These defects enhance the material's redox potential, promoting the generation of reactive oxygen species (ROS) that are essential for photocatalytic and Fenton-like degradation processes. Collectively, these physicochemical attributes govern the efficacy, reusability, and environmental compatibility of carbon–metal nanocomposites, rendering them highly versatile for the remediation of pharmaceutical contaminants.^59^

## 6 Mechanisms of pharmaceutical pollutant remediation

**Fig. 4:**
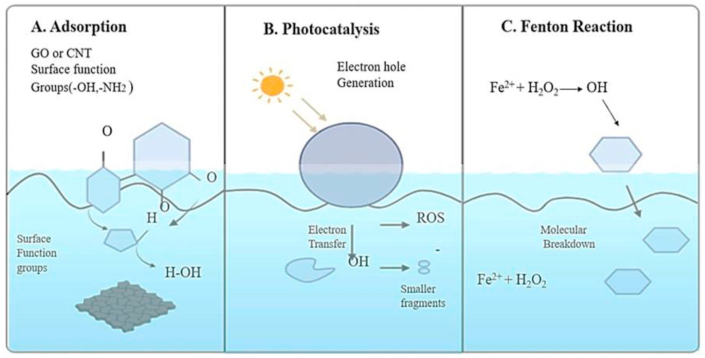
Mechanisms of pharmaceutical contaminants removal.

The remediation of pharmaceutical pollutants using carbon–metal nanocomposites relies on a combination of physicochemical mechanisms, each contributing to the adsorption, transformation, or degradation of complex drug molecules in aquatic systems. Fig. 4 illustrates the typical pollutant removal pathways mediated by carbon-based materials and metal oxides:

(A) Adsorption: Pharmaceuticals are captured on graphene oxide or carbon nanotube surfaces functionalized with hydroxyl and amine groups, facilitating strong intermolecular interactions.
(B) Photocatalysis: Light-induced generation of electron–hole pairs promotes the formation of reactive oxygen species (ROS), which oxidatively degrade organic contaminants.
(C) Fenton Reaction: In the presence of Fe^2+^ and H_2_O_2_, highly reactive hydroxyl radicals (•OH) are generated, driving the oxidative breakdown of pharmaceutical molecules.
These mechanisms exploit the structural, catalytic, and redox properties of both carbonaceous and metallic components, operating individually or synergistically to achieve efficient and selective pollutant removal.^60^

### 6.1 Adsorption and removal mechanisms

Adsorption serves as a primary mechanism for the initial removal of pharmaceutical contaminants from aqueous systems. Carbon-based materials—including graphene oxide, carbon nanotubes, and carbon dots—are especially effective due to their high specific surface areas and the abundance of functional groups. These materials interact with pharmaceutical molecules through a combination of π–π stacking, electrostatic interactions, hydrogen bonding, and van der Waals forces, enabling efficient and robust adsorption across a broad spectrum of contaminants.^61^ The effectiveness of these adsorption interactions is strongly dependent on the molecular structure of the pharmaceutical. Aromatic compounds, such as diclofenac and carbamazepine, interact preferentially with the sp^2^-hybridized carbon domains through π–π stacking, whereas polar or ionizable functional groups in drug molecules form hydrogen bonds or electrostatic interactions with surface moieties such as carboxyl, hydroxyl, or amine groups.^62^

Environmental parameters, including pH and temperature, markedly affect the adsorption efficiency of carbon nanomaterials by modulating both the ionization state of pharmaceutical contaminants and the surface charge of the adsorbent. Careful optimization of these conditions is therefore essential to achieve maximal removal of pharmaceutical residues under realistic environmental settings.^63^ Furthermore, the incorporation of metal nanoparticles onto carbon supports introduces additional active sites, enhancing the affinity of the material for pharmaceutical contaminants. These metal–carbon interfaces function as pre-concentration zones, bringing pollutants into close proximity with catalytic centers and thereby facilitating subsequent oxidative or photocatalytic degradation. This synergy between adsorption and catalysis highlights the critical role of surface engineering in the design of multifunctional nanomaterials for advanced water treatment applications.^64^ These adsorption interactions are schematically depicted in Fig. 4, which highlights the critical roles of surface functional groups and pore architecture in capturing pharmaceutical contaminants.

### 6.2 Catalytic degradation of pharmaceuticals

Metallic and metal oxide nanoparticles, including Fe_3_O_4_, ZnO, and Ag, serve as highly effective catalysts for the degradation of pharmaceutical contaminants via redox and oxidative pathways. These nanoparticles mediate degradation either directly through electron transfer reactions or indirectly by generating reactive species, particularly hydroxyl radicals (•OH) and superoxide anions (O_2_•^-^), which oxidize and break down complex pharmaceutical molecules. Iron-based nanomaterials are especially significant in this regard, as they catalyze Fenton and Fenton-like reactions; in the presence of hydrogen peroxide, they produce highly reactive hydroxyl radicals that enable rapid and extensive oxidation of organic pollutants.^65^ For example, silica-coated iron oxide nanocrystals subjected to microwave-assisted Fenton-like conditions have achieved degradation efficiencies exceeding 98% for methyl orange and over 91% for benzoic acid within minutes. The synergistic combination of microwave irradiation and acidic pH promotes enhanced radical generation and accelerates reaction kinetics, while the silica coating stabilizes the catalyst, minimizes nanoparticle aggregation, and preserves catalytic activity over multiple reuse cycles.^66^

Similarly, silver and copper nanoparticles display strong redox-cycling capabilities that promote dehalogenation, hydroxylation, and oxidative cleavage of pharmaceutical molecules. These catalytic activities are often enhanced when the nanoparticles are supported on carbon-based materials such as carbon nanotubes or graphene oxide. The carbon matrix mitigates nanoparticle agglomeration, improves electron transfer, and increases accessibility to active sites, thereby augmenting the overall catalytic efficiency of the nanocomposite.^67^ This synergistic interaction between metal catalysts and carbon nanomaterials leads to enhanced pollutant removal through both adsorption and degradation pathways, making them highly suitable for integrated water treatment applications targeting pharmaceutical residues.^68^

### 6.3 Electrochemical and photocatalytic processes

Carbon–metal nanocomposites are increasingly recognized for their dual functionality in electrochemical and photocatalytic degradation of persistent pharmaceutical contaminants. In electrochemical systems, these composites serve as electrodes or electrode modifiers, facilitating efficient electron transfer and reducing energy consumption. For example, sulfur-functionalized graphene sponge electrodes have achieved over 95% removal of sulfamethoxazole, alongside a 4.5-log reduction in bacterial counts, at a low energy input of 1.1 kWh/m^3^. This performance highlights the potential of carbon–metal hybrids for simultaneous pollutant degradation and microbial disinfection.^69^

The synergistic interplay between carbon-based materials and metal or metal oxide components underpins these remediation processes. Carbon nanostructures, including graphene, carbon nanotubes, and carbon dots (CDs), provide high electrical conductivity and extensive surface area, facilitating efficient charge transport and access to catalytic sites. Concurrently, semiconducting metal oxides such as TiO_2_, ZnO, and doped Fe_2_O_3_ function as photocatalysts under UV or visible light. Light excitation generates electron–hole pairs that react with water and dissolved oxygen to form reactive oxygen species (ROS), including hydroxyl radicals (•OH) and superoxide anions (•O_2_^−^). These ROS drive the oxidative degradation of complex pharmaceutical molecules, transforming them into less toxic or more biodegradable intermediates.^70^

Carbon dots further enhance photocatalytic performance through their intrinsic up-conversion photoluminescence, enabling the utilization of lower-energy visible light for reactive oxygen species (ROS) generation. Their abundant surface functional groups promote strong pollutant binding and intimate contact with the catalytic interface. By integrating adsorption, redox catalysis, and light-driven degradation, carbon–metal nanocomposites serve as versatile and highly efficient platforms for advanced water purification, specifically targeting pharmaceutical contaminants.^5^

### 6.4 Synergy between carbon and metal components in pollutant degradation

The integrated design of carbon–metal nanocomposites generates a pronounced synergistic effect that significantly enhances the efficiency of pharmaceutical pollutant remediation. Carbon-based components, including graphene, carbon nanotubes, and carbon dots, provide high surface area, excellent dispersibility in aqueous media, and strong adsorption affinity toward contaminants. Concurrently, embedded metal or metal oxide nanoparticles impart catalytic and redox functionalities, facilitating the generation of reactive oxygen species (ROS) and promoting advanced oxidation processes. The intimate interfacial contact between the carbon and metal phases is crucial for rapid electron transfer, reduction of charge recombination during photocatalysis, and stabilization of active catalytic sites.^71^

For instance, in a CuO/CoFe_2_O_4_/multi-walled carbon nanotube (MWCNT) nanocomposite, the MWCNTs provide efficient adsorption sites for tetracycline, while CuO and CoFe_2_O_4_ function as active photocatalytic components under visible light. The integrated architecture enhances electron mobility and suppresses charge recombination, thereby accelerating degradation kinetics and improving overall removal efficiency. This close interfacial interaction not only increases the degradation rate but also reduces the energy input required and extends the operational lifetime of the material by improving stability and reusability.^72^

These hybrid systems frequently exhibit broader efficacy against diverse pharmaceutical contaminants compared with their individual components, rendering them especially suitable for multifunctional water purification strategies. By integrating adsorption, catalytic degradation, and, in some cases, antimicrobial activity, carbon–metal nanocomposites provide a robust, scalable, and sustainable platform for advanced water treatment applications.^73^

## 7 Carbon metal nanocomposites for pharmaceutical remediation

Carbon nanotubes (CNTs) provide an excellent platform for nanocomposite construction owing to their high aspect ratio, remarkable tensile strength, and large surface area. Their hollow tubular morphology offers both internal and external adsorption sites, enabling efficient capture of linear and aromatic pharmaceutical contaminants. When decorated with metal or metal oxide nanoparticles, such as silver (Ag), zinc oxide (ZnO), or iron oxide (Fe_3_O_4_), CNTs transform into multifunctional materials capable of both physical adsorption and catalytic degradation.^74^

The incorporation of iron oxides into CNTs significantly enhances their catalytic performance, particularly in Fenton-like reactions, by facilitating the generation of hydroxyl radicals (•OH) for oxidative degradation. For example, nano-iron oxide supported within a CNT-based flow-through membrane achieved over 98% removal of tetracycline under mildly acidic conditions. This high efficiency is attributed to the synergistic interplay between the extensive surface area of CNTs and the redox catalytic activity of iron oxides, rendering these composites highly effective for advanced water purification applications.^75^

**Fig. 5:**
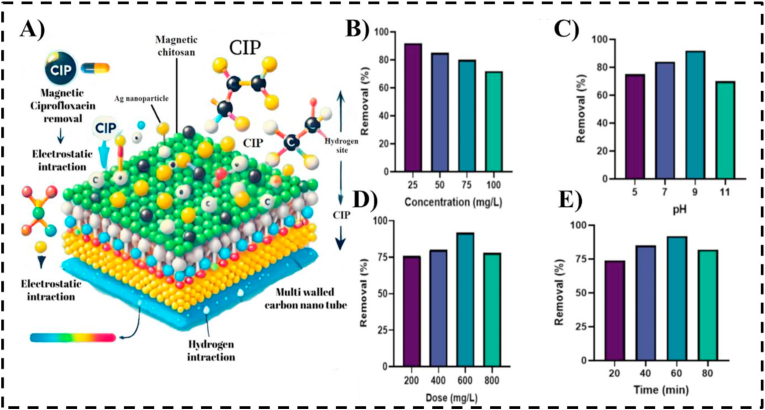
A) Schematic illustration of ciprofloxacin (CIP) removal mechanism using a magnetic chitosan–Ag nanoparticle–multi-walled carbon nanotube (MWCNT) composite. (B–E) Effects of various operational parameters on ciprofloxacin removal efficiency, including (B) initial CIP concentration, (C) pH, (D) adsorbent dosage, and (E) contact time.^76^

Silver nanoparticle-functionalized CNTs provide additional bactericidal functionality, enabling simultaneous mitigation of chemical contaminants and microbial hazards. When incorporated into a magnetic chitosan matrix, Ag-decorated multi-walled CNTs effectively adsorbed ciprofloxacin, a commonly detected antibiotic and a contributor to antimicrobial resistance in wastewater. The composite architecture—comprising chitosan, silver nanoparticles, and CNTs—facilitates electrostatic interactions, hydrogen bonding, and π–π stacking, while the magnetic core allows facile recovery and reuse. Adsorption behavior followed the Langmuir model, with a maximum capacity of 31.26 mg/g, underscoring the influence of structural design on adsorption efficiency. Fig. 5A schematically illustrates the ciprofloxacin removal mechanism using this magnetic chitosan–Ag nanoparticle–MWCNT composite, highlighting electrostatic and hydrogen bonding interactions, while Fig. 5B–E depicts the effects of operational parameters—including initial CIP concentration, pH, adsorbent dosage, and contact time—on removal efficiency.^76^ Overall, CNT–metal nanocomposites integrate adsorption, catalytic degradation, and antimicrobial functionalities within a single platform. This multifunctionality, combined with tunable surface chemistry and structural design, highlights their potential as advanced materials for the efficient removal of pharmaceutical pollutants from complex aqueous environments. Table 2 summarizes representative carbon nanotube–metal nanocomposites and their applications in pharmaceutical pollutant remediation.

**Table 2:** Carbon Nanotube Metal Nanocomposites for pharmaceutical remediation.

Metal/Metal Oxide	Target Pharmaceuticals	Synthesis Method	Removal Efficiency	Remediation Mechanism	Experimental Conditions	Reference
MWCNT/Fe3O4	Naproxen	Co-Precipitation Method	82%	Adsorption	pH = 5, T = 70 °C, 10.0 mg/L	77
MWCNT/Cobalt oxide nanoparticles (Co_3_O_4)_	Oxytetracycline (OTC)	Pyrolysis–Oxidation	93.6% (14.04 mg g1 h1)	Adsorption-desorption	pH = 3.0 to 9.0, T = 25 °C,20 mg L1)	78
MWCNT/Cu Fe_2_O_3_	Trimethoprim	Sol–Gel Combustion	89%	oxidative degradation	pH = 7.0, T = 27 °C, 0.02 mM	79
MWCNT/MnO_2_/Fe_3_O_4_	Ibuprofen And Paracetamol	Hydrothermal Method	85.6%, 89.7 %	Adsorption	pH = 2, 1 g L−1, T = 20 °C, 20 min	80
MWCNT/ZnO/spinel cobalt ferrite (CoFe2O4)	Cefixime	Co-Precipitation	100%	Adsorption	PH = 7.0, 10 mg/L,20min, UV	81
MWCNT/Tungsten oxide (WO3)	Tetracycline	Sol-Gel/Hydrothermal	83.7%	Sono-Photocatalytic Degradation	pH = 9.0,0.7 g/L, 60 min, ultrasound (US) irradiations	82
MWCNT/Nickel-copper ferrite nanoparticles (CuNiFe2O4))	Ampicillin	Coprecipitation	92.42%	Photocatalytic degradation	pH = 5, 0.5 g/L, 60 min	83
MWCNT/Spinel nickel ferrite (NiFe2O4)	Sulfamethoxazole	Hydrothermal	68%	Photodegradation	pH = 1.7,5 mg/L, UV	84
MWCNT/Mn-WO_3_	Diclofenac	Hydrothermal Doping	88%	Photocatalytic degradation	pH = 7, 2.2 g/L catalyst, 70–180 min.	
MWCNT/Ag/Fe_3_O_4_	Ciprofloxacin	In situ impregnation	94% (31.26 mg/g)	Adsorption (langmuir model)	pH = 7, 50 mg/L CIP, 0.5 g/L nanocomposite, 60 min	76

### 7.1 Graphene oxide metal nanocomposites

**Fig. 6:**
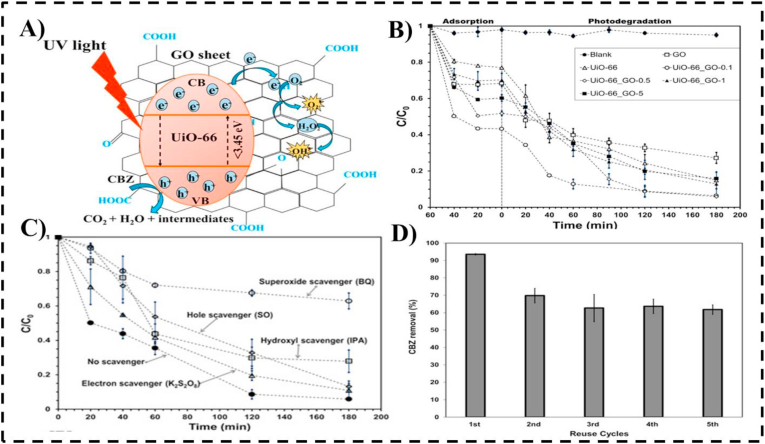
(A) Photocatalytic degradation mechanism of carbamazepine (CBZ) using a UiO-66/graphene oxide (GO) composite under UV light irradiation, illustrating charge transfer, reactive oxygen species (ROS) generation, and CBZ mineralization. (B) Photodegradation performance comparison for CBZ using different UiO-66/GO composite ratios. (C) Reactive species trapping experiments confirming the involvement of superoxide radicals, holes, hydroxyl radicals, and electrons in CBZ degradation. (D) Reusability test of the UiO-66/GO composite over five consecutive cycles, demonstrating its stability and sustained CBZ removal efficiency.^89^.

Graphene oxide (GO) serves as a highly versatile platform for environmental remediation due to its two-dimensional sheet-like morphology, abundant oxygen-containing functional groups (e.g., hydroxyl, carboxyl, epoxy), and exceptional electron mobility. These features enable strong interactions with diverse pharmaceutical pollutants through electrostatic attraction and π–π stacking, while also providing active sites for the immobilization of metal or metal oxide nanoparticles. The decoration of GO with nanoparticles such as zinc oxide (ZnO) and copper oxide (CuO) markedly enhances photocatalytic degradation performance, particularly under visible light. For example, GO-coated CuO nanostructures achieved up to 95% degradation of diclofenac within 60 min of irradiation. This high efficiency arises from the synergistic interplay between GO's large surface area and the catalytic activity of CuO, where interfacial contact promotes rapid electron transfer, suppresses charge recombination, and stabilizes active catalytic sites.^85^ Beyond enhancing light-driven degradation, the oxygen-rich surface chemistry of GO promotes strong coordination with metal ions, ensuring uniform nanoparticle dispersion and structural stability. This renders GO-based composites particularly effective against persistent pharmaceutical contaminants, including hormones and antiepileptic drugs, which are often refractory to conventional treatment. The integration of adsorption and photocatalysis within these nanocomposites highlights GO's potential as a dual-function scaffold for advanced water purification technologies.^86^, ^87^, ^88^ UiO-66/graphene oxide (UiO-66–GO) nanocomposites synthesized via a one-step hydrothermal method exhibited significantly enhanced photocatalytic degradation of carbamazepine (CBZ), achieving over 90% removal within 2 h. The composite displayed a maximum rate constant of 0.0136 min^−1^, which was 2.8- and 1.7-fold higher than those of pristine GO and UiO-66, respectively. This improved performance was attributed to increased surface area, enhanced light absorption, and a reduced band gap. Moreover, the nanocomposite demonstrated good stability over five consecutive reuse cycles, highlighting its potential for practical water treatment applications.^89^ Fig. 6 illustrates the key aspects of the UiO-66/GO composite system: (A) Schematic representation of the photocatalytic degradation mechanism of carbamazepine (CBZ) using a UiO-66/graphene oxide (GO) composite under UV irradiation, highlighting charge transfer, reactive oxygen species (ROS) generation, and subsequent CBZ mineralization. (B) Comparison of CBZ photodegradation efficiency for different UiO-66/GO composite ratios, demonstrating the effect of composition on performance. (C) Reactive species trapping experiments, confirming the roles of superoxide radicals (•O_2_^−^), photogenerated holes (h^+^), hydroxyl radicals (•OH), and electrons (e^−^) in CBZ degradation. (D) Reusability assessment of the UiO-66/GO composite over five consecutive cycles, indicating stable photocatalytic activity and sustained CBZ removal efficiency.^89^ Graphene oxide–metal nanocomposites have demonstrated significant potential for the removal of pharmaceutical pollutants, owing to their high surface area, abundant functional groups, and synergistic interactions with embedded metal nanoparticles (Table 3).

**Table 3:** Graphene Oxide Metal Nanocomposites for pharmaceutical remediation.

Metal/Metal Oxide	Target Pharmaceuticals	Synthesis Method	Removal Efficiency (%)	Remediation Mechanism	Experimental Conditions	Reference
GO/Zirconium	carbamazepine (CBZ)	hydrothermal method	90%	photocatalytic oxidation	pH = 7, 5 mg/L, 2 h	89
GO/ZnO	Steroid estrogens	microwave assisted	89%	Photodegradation	pH 5.0, 10 min	90
GO/TiO_2_/Pt	Tetracycline	Pyrolytic route	∼81%	Photocatalytic degradation	pH ≈ 7, 45 °C, visible light	91
GO/NiFe_2_O_4_/CeO_2_	Tetracycline HCl	Hydrothermal method	95%	Photocatalytic degradation	Visible light, 90 min	42
GO/β-Cyclodextrin (β-CD)	Chloramphenicol, SMZ, Diclofenac	Hydrothermal method	92–96%	Adsorption via H-bonding & π–π stacking	pH 4–8, 6 h contact time	92
GO/Nb-doped ZnO	Tetracycline	Green synthesis using plant extract	88%	Photocatalytic degradation	Visible light, 75 min	93
GO/ZnO/Ag	Ciprofloxacin	Sol–gel method	98%	Photocatalysis	UV light, pH 5, 60 min	94
GO/Fe_3_O_4_	Ibuprofen	Co-precipitation	90%	Adsorption	pH 5.5, 180 min	95
GO/Fe/Mgo	Rhodamine B	hydrothermal	86%	Photocatalytic degradation	Solar light 60 min	96

### 7.2 Carbon dot metal nanocomposites

**Fig. 7:**
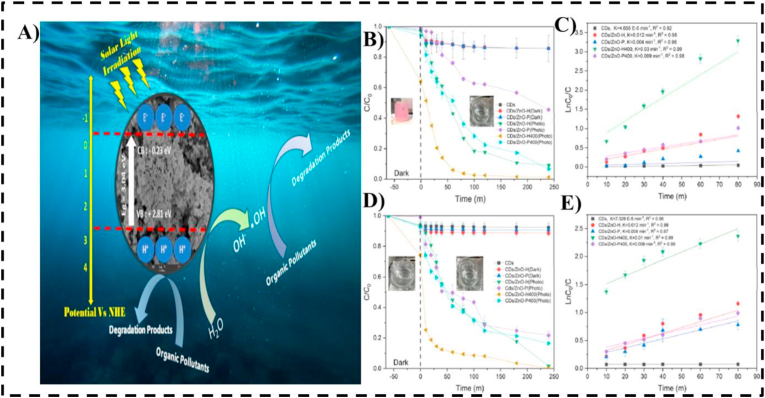
(A) Schematic illustration of the photocatalytic degradation mechanism of organic pollutants using CdZnO-based nanocomposite under solar light irradiation, highlighting electron–hole pair generation, hydroxyl radical formation, and pollutant mineralization. (B, C) Photocatalytic degradation efficiency and corresponding kinetic plots for Congo Red (CR) under various experimental conditions. (D, E) Photocatalytic degradation efficiency and kinetic plots for Rhodamine B under different conditions, demonstrating the enhanced photocatalytic activity of the CdZnO-based composite.^101^

Carbon dots (CDs) are quasi-spherical, photoluminescent nanostructures distinguished by excellent aqueous dispersibility, abundant surface functionalities, and tunable photophysical properties. Their integration with metal oxides such as TiO_2_, ZnO, and CuO produces hybrid photocatalysts capable of degrading trace-level pharmaceutical pollutants under both UV and visible light. Serving as efficient light sensitizers, CDs enhance the photocatalytic activity of metal oxides by promoting electron transfer, suppressing charge recombination, and extending light absorption into the visible spectrum. These features render CD-based composites particularly effective against recalcitrant pharmaceutical compounds, including carbamazepine, sulfamethoxazole, and estradiol.^97^ A magnetically recoverable chitosan–carbon quantum dot/ZnFe_2_O_4_ nanocomposite has been developed for the simultaneous removal of tetracycline antibiotics and associated antibiotic resistance genes (ARGs) from wastewater, integrating adsorption with visible-light-driven photocatalysis. The nanocomposite achieved near-complete tetracycline degradation and substantial ARG attenuation while maintaining high reusability owing to its magnetic separability. These results highlight the strong potential of carbon dot–metal nanocomposites as efficient, regenerable platforms for advanced pharmaceutical wastewater treatment.^98^ The construction of heterojunction photocatalysts, exemplified by carbon-doped g-C_3_N_4_/TiO_2_ systems, demonstrates the synergistic enhancement of adsorption and photocatalytic activity. These composites have achieved degradation efficiencies exceeding 98% for pharmaceutical compounds such as diclofenac and carbamazepine under LED illumination. The layered architecture, combined with carbon doping, promotes efficient charge separation and facilitates the generation of reactive oxygen species (ROS), thereby accelerating the oxidative breakdown of complex pharmaceutical structures.^99^ Up-conversion luminescence, a hallmark property of carbon dots (CDs), enables the conversion of longer-wavelength light into higher-energy emissions, effectively activating metal oxide photocatalysts under ambient light conditions. Surface passivation with heteroatoms such as nitrogen or sulfur further enhances the binding affinity of CDs for pharmaceutical molecules and improves their electron donor capability. For example, a cost-effective photocatalyst, CD/ZnO-H400, was synthesized using carbon dots derived from coconut air and ZnO via hydrothermal treatment (180 °C, 4 h) followed by calcination (400 °C, 4 h). This composite exhibited high degradation efficiencies for RR141 dye (98%) and ofloxacin (96%) under sunlight, demonstrating its potential as a sustainable and efficient photocatalytic platform for water purification.^100^ The photocatalytic degradation by CD/ZnO-H400 followed first-order kinetics, with rate constants of 0.03 min^−1^ for RR141 and 0.01 min^−1^ for ofloxacin. The catalyst maintained its activity over five reuse cycles, demonstrating excellent stability and solar-driven detoxification potential. Fig. 7 illustrates (A) the photocatalytic mechanism of the CD–ZnO nanocomposite under solar irradiation, including electron–hole pair generation, hydroxyl radical formation, and pollutant mineralization; (B, C) the degradation efficiency and kinetic plots for Congo Red (CR) under various experimental conditions; and (D, E) the corresponding data for Rhodamine B, highlighting the enhanced photocatalytic performance of the composite.

Carbon dot–metal nanocomposites have thus emerged as highly promising materials for pharmaceutical remediation due to their strong photoluminescence, abundant surface functionalities, and synergistic interactions with metal nanoparticles (Table 4).

**Table 4:** Carbon Dot Metal Nanocomposites for pharmaceutical remediation.

Metal/Metal Oxide	Target Pharmaceuticals	Synthesis Method	Removal Efficiency (%)	Remediation Mechanism	Experimental Conditions	Reference
CD/Titanium dioxide (TiO2)	Caffeine, carbamazepine, and Ibuprofen	hydrothermal method	72.66%	photocatalysis	Time = 6 h,0.79 mg/L	102
CD/Titanium dioxide (TiO2)	Sulfadiazine, Sulfamethoxazole and Trimethoprim	Hydrothermal method	90%	Photodegradation	Time-0.3 h,500 mg L^−1^	103
CD/TiO2	Oxolinic acid	hydrothermal process	85-91%	photocatalysis	pH = 7.3500 mg/L, 40 min	104
CD/Zinc oxide	Ofloxacin antibiotics	hydrothermal methods	96%	Photoactivity	pH = 7	101
CD/TiO2	Sulfadiazine (SDZ) and Oxolinic acid (OXA)	hydrothermal treatment	90%	photodegradation	22 min,500 mg L^−1^	105
CQD/ZnO	Methylene blue	Electrooxidation of ethanol	90%	Photodegradation	pH 7, 5 min, visible light	106
CD/ZnO_1-x_S_x_	Methyl blue dye	Pulsed electrodeposition and annealing	92%	Photocatalysis	pH 6, 30 min, visible light	106
Nitrogen-doped CQDs/ZnO nanorods	Rhodamine B	Hydrothermal method	90%	Photodegradation	pH 7, 9 min, UV light	107
CD/Titanate nanotubes (TNTs)	Caffeine	One-pot hydrothermal synthesis	85%	Photocatalysis	pH 6.5, 90 min, visible light	108

## 8 Environmental and ecotoxicological considerations

In addition to direct toxicity, the environmental fate and transport of carbon–metal nanocomposites are influenced by aggregation, sedimentation, and interactions with natural organic matter, which can alter their reactivity and bioavailability. Metal leaching from nanocomposites, particularly from silver, copper, or iron-based systems, may contribute to heavy metal contamination and exert additional toxic effects on microbial and aquatic communities. Furthermore, the long-term persistence of these nanomaterials in surface waters and sediments could lead to chronic exposure scenarios, affecting reproductive, developmental, and physiological processes in sensitive organisms.Overall, while carbon–metal nanocomposites offer powerful remediation capabilities, their environmental release necessitates careful risk assessment, lifecycle analysis, and the development of safe handling, recovery, and disposal strategies to mitigate ecotoxicological impacts.^109^ Adverse effects on non-target organisms represent an additional major concern. Although carbon nanomaterials like carbon nanotubes (CNTs) and graphene oxide are efficient in removing pharmaceuticals, their potential impacts on microorganisms, plants, and benthic species are still not well understood.^110^ The type of metal nanoparticles incorporated significantly affects toxicity, with metals like silver and copper exhibiting antimicrobial effects that can disrupt aquatic microbial communities. In a study by Pikula et al., the toxicity of multiwalled CNTs, graphene oxide, graphene, and fullerene (C_60_) was assessed against the red microalga *Porphyridium purpureum*, yielding EC_50_ values of 2.08, 23.37, 94.88, and >131 mg/L, respectively. These results indicate that CNTs are markedly more toxic than other carbon nanomaterials, and notably, they were the only material to trigger substantial reactive oxygen species (ROS) generation.^111^ In addition, metal ion leaching from nanocomposites may further elevate risks to sensitive aquatic species.

Regulatory frameworks are becoming increasingly important for the safe deployment of carbon-based nanomaterials. The OECD has developed test guidelines covering physicochemical characterization, environmental fate, ecotoxicity, and human health impacts of manufactured nanomaterials. In the European Union, nanoforms are regulated under REACH, whereas in the United States, the EPA assesses nanoscale materials through statutes such as TSCA and the Safe Drinking Water Act. In India, the Central Pollution Control Board is starting to incorporate considerations for nano-enabled materials within its pharmaceutical effluent guidelines.^112^ Although specific discharge limits for most carbon-based nanocomposites are not yet established, current regulations emphasize minimizing metal leaching, ensuring material recoverability, and implementing clear end-of-life management practices. Additionally, thorough ecosystem risk assessments should be incorporated into regulatory frameworks, addressing both acute toxicity and long-term impacts, including bioaccumulation, trophic transfer, and broader ecological disturbances.^113^ The design of sustainable nanocomposites should focus on biodegradability, controlled metal release, and the minimization of secondary pollution. Raza et al. highlighted the importance of integrating environmental risk assessments into the development of biodegradable nanocomposites, pointing out bioaccumulation and trophic transfer as critical long-term considerations and recommending the use of eco-friendly metals and materials to reduce ecological impacts.^114^

## 9 Challenges and limitations

Despite their strong promise, translating carbon–metal nanocomposites from laboratory studies to large-scale water treatment faces significant hurdles. Scalability is a primary concern: techniques like chemical vapor deposition, hydrothermal synthesis, and sol-gel processes, while effective at small scale, are often resource- and time-intensive and can compromise structural consistency when scaled up. Preserving the nanocomposite's structural and functional integrity during scale-up is crucial for dependable field performance. Solid-state blending methods have emerged as viable alternatives; for example, TiO_2_/rGO composites with optimized rGO content achieved 95.18% photodegradation of acetaminophen under UV light within 100 min, maintaining catalytic efficiency across multiple reuse cycles. These results underscore the need for scalable synthesis strategies that deliver both high performance and long-term stability.^115^ Another critical challenge is understanding the environmental fate and behavior of carbon–metal nanocomposites after their introduction into aquatic systems. Interactions with natural organic matter (NOM), co-existing pollutants, and microbial communities can substantially modify their physicochemical properties. In natural waters, these nanocomposites may undergo aggregation, surface passivation, or redox transformations, which can decrease their contaminant-removal efficiency or produce unintended ecological effects. For example, fulvic acids—a major component of NOM—have been reported to reduce adsorption efficiency by 45–60% and slow photodegradation rates by approximately 25%, largely due to surface coverage and inhibited electron transfer. These observations highlight the importance of accounting for matrix effects when developing nanocomposites for practical water treatment applications.^116^

Economic and sustainability factors present additional barriers to the large-scale deployment of carbon–metal nanocomposites. The fabrication of high-quality carbon materials and metal nanoparticles often involves costly precursors and energy-intensive processes. While green synthesis approaches are emerging, challenges remain in achieving consistent scalability and reproducibility. Dependence on precious or potentially toxic metals, such as silver or gold, further raises ecological and sustainability concerns. Metal leaching can reduce long-term efficacy and introduce secondary contamination into aquatic ecosystems. Therefore, future efforts should prioritize the development of cost-effective, eco-friendly, and recyclable nanocomposites that minimize environmental impact throughout their lifecycle.^117^ Compared to many purely metal-oxide or noble-metal systems, carbon-based nanocomposites present distinct advantages in terms of cost-effectiveness and environmental sustainability. Carbon frameworks can be produced from abundant, low-cost sources such as biomass, agricultural residues, or industrial carbon wastes. Green synthesis methods—including hydrothermal carbonization or bio-derived reducing agents—further reduce energy consumption and hazardous chemical use. Their high surface area and tunable chemistry enhance adsorption capacity and, in photocatalytic hybrids, improve light utilization, lowering both material dosage and energy requirements per unit of contaminant removed. Additionally, magnetic or structurally robust carbon–metal composites allow repeated recovery and regeneration with minimal performance loss, spreading material and energy costs across multiple cycles and reducing secondary waste compared with single-use adsorbents or homogeneous oxidants.

Lastly, regulatory ambiguity remains a significant barrier. Existing environmental and health frameworks seldom account for the distinctive properties and risks of engineered nanomaterials. Key factors—including bioaccumulation potential, ecological persistence, and nanoparticle-specific toxicities—are still poorly characterized and lack formal oversight. The absence of standardized risk assessment protocols generates uncertainty in commercialization and restricts large-scale application. Establishing comprehensive safety guidelines, robust environmental monitoring, and nanomaterial-specific regulations is therefore crucial to enable responsible and safe deployment.^118^ Together, these challenges highlight the importance of multidisciplinary research and policy integration to advance carbon–metal nanocomposites from promising lab-scale materials to practical, safe, and sustainable solutions for pharmaceutical pollutant remediation.^119^

## 10 Economic feasibility and sustainability

A thorough evaluation of carbon–metal nanocomposites for pharmaceutical remediation must extend beyond laboratory performance and consider production costs, life-cycle impacts, recyclability, and energy requirements. Production expenses are largely dictated by the choice of carbon source—fossil-derived versus biomass-derived—the synthesis method (CVD, hydrothermal, sol–gel), and the type of metal used. High-temperature CVD and noble-metal incorporation generally incur greater capital and operating costs compared with low-temperature, bio-based approaches.

Life-cycle assessments highlight that energy-intensive synthesis, operational demands such as pumping or irradiation, and end-of-life management significantly affect the environmental footprint, emphasizing the need for energy-efficient synthesis, durable materials, and closed-loop regeneration strategies. Reusability and recyclability are critical for economic viability: although many carbon-based nanocomposites retain over 80–90% of their initial adsorption or photocatalytic performance across multiple cycles, fouling, structural degradation, or metal leaching can reduce service life and elevate replacement and waste management costs.

**Fig. 8:**
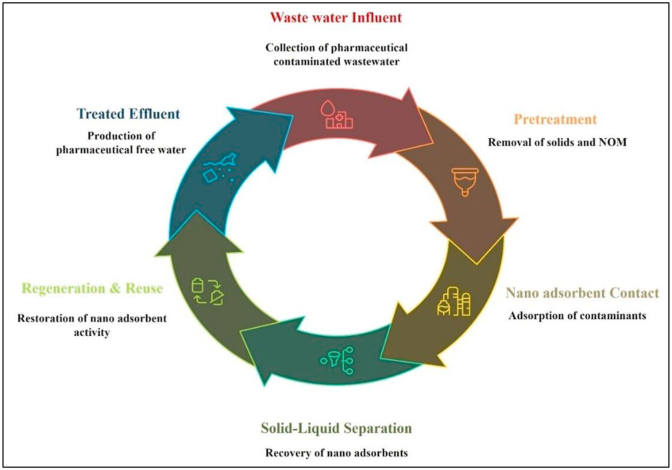
Conceptual Large-Scale Application of Nano adsorbents for Pharmaceutical Wastewater Treatment.

From an energy standpoint, integrating nanocomposites into low-pressure adsorption columns, passive solar-driven photocatalytic reactors, or magnetically recoverable batch systems can lower energy consumption relative to intensive processes like high-pressure membranes or aeration-driven advanced oxidation. However, quantitative techno-economic metrics—such as cost per cubic meter treated or kWh per cubic meter—remain scarce, representing a key research gap for sustainable scale-up. Fig. 8 illustrates a conceptual framework for implementing nano-adsorbents in large-scale pharmaceutical wastewater treatment systems.

## 11 Future perspectives and research directions

The future of carbon–metal nanocomposites in pharmaceutical remediation hinges on enhancing efficiency, sustainability, and scalability. One promising approach is the design of hybrid nanocomposites that combine carbon materials with novel metals or advanced functional components. Fine-tuning the physicochemical properties of both the carbon matrix and the metal nanoparticles increases selectivity and reactivity toward pharmaceutical contaminants. Incorporating photocatalytic or magnetic functionalities has proven effective for superior pollutant removal under environmentally relevant conditions, while green-synthesized metals and benign functional groups support large-scale, eco-friendly deployment.

Integration with complementary remediation technologies—such as bioremediation, membrane filtration, and advanced oxidation processes (AOPs)—further expands functionality, enabling treatment of complex water matrices containing diverse pharmaceuticals. For example, ZnO–graphene and Fe_3_O_4_–CNT hybrids in AOP–adsorption systems have shown improved degradation efficiency while minimizing secondary pollutant formation, making them suitable for decentralized or in situ water treatment applications.

Natural organic matter (NOM) can hinder adsorption or photocatalytic performance by 45–60%, but strategies such as surface functionalization with hydrophilic, zwitterionic, amine, or thiol groups, heteroatom doping for charge tuning, and the use of antifouling inorganic components or core–shell architectures can mitigate these effects. Process-level measures—including magnetic recovery and light-driven self-cleaning—further enhance long-term stability in NOM-rich waters.

Sustainability is reinforced through green synthesis routes using plant extracts, microbial agents, or biodegradable precursors. Magnetic carbon-based composites derived from chitosan or agricultural-waste silica exhibit high adsorption capacity, reusability, and facile magnetic separation, demonstrating the synergy of functional performance with eco-friendly design.For industrial-scale application, optimized continuous manufacturing processes and collaboration among academia, industry, and regulatory bodies are essential. With the growing global challenge of pharmaceutical contamination, carbon–metal nanocomposites offer a scalable, high-performance, and environmentally responsible solution for next-generation water treatment systems.

## 12 Conclusion

Carbon–metal nanocomposites represent a versatile and promising platform for the sustainable remediation of pharmaceutical pollutants in aquatic environments. Their high surface area, tunable surface chemistry, and multifunctional capabilities enable efficient adsorption, catalytic degradation, and antimicrobial activity, addressing the complex nature of pharmaceutical contamination. Experimental studies have demonstrated their effectiveness across a broad spectrum of drug molecules, yet comprehensive evaluations of long-term environmental impacts—including toxicity, stability, and persistence—remain necessary.

Future research should prioritize scalable, cost-effective, and green synthesis strategies, alongside advanced functionalization to enhance selectivity, reactivity, and resistance to fouling by natural organic matter. Integration with complementary remediation technologies, such as advanced oxidation processes, membrane filtration, and bioremediation, can further expand their applicability in real-world water treatment scenarios. By coupling high performance with environmentally responsible design and careful risk assessment, carbon–metal nanocomposites hold significant potential for large-scale deployment, offering a sustainable solution to pharmaceutical pollution and contributing to the advancement of next-generation water treatment systems.

## CRediT authorship contribution statement

**Deepshikaa Kannan:** Writing – original draft. **Bellarmin Michael:** Writing – review & editing, Writing – original draft, Data curation. **Elizabethrani Edwin:** Writing – review & editing, Conceptualization. **Jayashree Srinivasan:** Writing – review & editing. **Sakthi Sanjana:** Writing – review & editing. **Nandhini Jayaprakash:** Writing – review & editing, Supervision.

## Declaration of generative AI and AI-assisted technologies in the writing process

During the preparation of this work, the authors used Quill Bot and Paper pal for enhancing language, flow, and readability. After utilizing these tools, the authors reviewed and edited the content as necessary and takes full responsibility for the final content.

## Funding

No Funding.

## Declaration of competing interest

The authors declare that they have no known competing financial interests or personal relationships that could have appeared to influence the work reported in this paper.

## References

[1] Amin M.F., Rahman M.S. (2026). A critical review of pharmaceutical pollutants in soil and air: ecotoxicological impacts on animal, plant and microbial communities - health hazards and waste management. Environ Pollut Manag.

[2] Shabnum S.S., Siranjeevi R., Raj C.K., Nivetha P., Benazir K. (2025). A comprehensive review on recent progress in carbon nanotubes for biomedical application. Environ Qual Manag.

[3] Mohd Hanafiah Z., Wan Mohtar W.H.M., Abdul Maulud K.N. (2025). Global pharmaceutical pollution in waterways: insights from sewage treatment point sources. Emerging Contam.

[4] Mahalakshmi D., J N., Karthikeyan E. (2025). Carbon nanomaterials for emerging contaminant remediation: addressing pharmaceutical pollution in the water cycle with precision. Water Cycle.

[5] Kaliyaperumal R., Nagaraj K., Kasilingam T., Kumaravel T., Gokulan R. (2025). Synthesis, characterization, and multifunctional bioactivity of cadmium oxide-modified graphitic carbon nitride (CdO/G-C3N4) nanocomposites for biomedical applications. BioNanoScience.

[6] Coderre M., Fortin A.S., Morency L.D., Roy J., Sirois C. (2025). Pharmaceuticals in drinking water: a scoping review to raise pharmacists' public health and environmental awareness on contamination in groundwater, surface water, and other sources. Int J Pharm Pract.

[7] da Silva R.L., Lima e, Silva M.A., Teixeira T.P. (2025). A systematic review of estrogens as emerging contaminants in water: a global overview study from the one health perspective. J Xenobiot.

[8] Tang Z., Liu Z hua, Wang H., Dang Z., Liu Y. (2021). A review of 17α-ethynylestradiol (EE2) in surface water across 32 countries: sources, concentrations, and potential estrogenic effects. J Environ Manag.

[9] Baraneedharan P., Vadivel S., C A.A., Mohamed S.B., Rajendran S. (2022). Advances in preparation, mechanism and applications of various carbon materials in environmental applications: a review. Chemosphere.

[10] Tsaridou C., Karabelas A.J. (2021). Drinking water standards and their implementation—A critical assessment. Water (Basel).

[11] Okuthe G.E., Dube E., Mafunda P.S. (2025). Effects of pharmaceuticals and endocrine-disrupting chemicals on reproductive biology of aquatic fauna: penguins as sentinel species. J Xenobiot.

[12] Srivastava S.K. (2024). Recent advances in removal of pharmaceutical pollutants in wastewater using metal oxides and carbonaceous materials as photocatalysts: a review. RSC Appl Interf.

[13] El Messaoudi N., Miyah Y., Benjelloun M. (2024). A comprehensive review on designing nanocomposite adsorbents for efficient removal of 4-nitrophenol from water. Nano-Struct Nano-Objects.

[14] Holilah Asranudin, El Messaoudi N. (2024). Fabrication a sustainable adsorbent nanocellulose-mesoporous hectorite bead for methylene blue adsorption. Case Stud Chem Environ Eng.

[15] Sdiq A.F.H., Abdulrahman H.H., Ismail H.K. (2025). A review of effective nanoadsorbents made of carbonaceous, metal oxides, and polymer nanocomposite materials for adsorption of pharmaceutical contaminants in wastewater. Int J Environ Res.

[16] Abdulrahman H.H., Sdiq A.F.H., Ismail H.K. (2025). Polymer nanocomposite adsorbents for the removal of pharmaceutical formulations the aquatic environment: a review. Water Air Soil Pollut.

[17] Abdulrahman H.H., Ismail H.K. (2025). Adsorption of metronidazole and tetracycline pharmaceuticals by Poly(3,4-ethylenedioxythiophene)/Magnetite@zinc ferrite (PEDOT/Fe3O4@ZnFe2O4) adsorbent synthesized from deep eutectic solvent: kinetic, isothermic, and thermodynamic studies. J Inorg Organomet Polym Mater.

[18] Chand S., Kumar R., Thakur N. (2025). Sustainable synthesis and multifunctional applications of biowaste-derived carbon nanomaterials and metal oxide composites: a review. Chemosphere.

[19] Ingale M.H., Tayade M.C., Patil Y.P., Salunkhe R.H. (2023). Pharmaceutical waste disposal current practices and regulations: review. Int J Pharm Qual Assur.

[20] Mahalakshmi D., J N., Karthikeyan E. (2025). Carbon nanomaterials for emerging contaminant remediation: addressing pharmaceutical pollution in the water cycle with precision. Water Cycle.

[21] Maity T., Kumar Y., Deb A.K.S., Ali S.M., Maiti P.K. (2025). Enhanced and Efficient Extraction of Uranyl Ions from Aqueous Waste Through Graphene/CNT-PAMAM Nanocomposites. Langmuir.

[22] Jayaprakash N., Elumalai K., Manickam S., Bakthavatchalam G., Tamilselvan P. (2024). Carbon nanomaterials: revolutionizing biomedical applications with promising potential. Nano Mater Sci.

[23] Kaymaz S.V., Nobar H.M., Sarıgül H., Soylukan C., Akyüz L., Yüce M. (2023). Nanomaterial surface modification toolkit: principles, components, recipes, and applications. Adv Colloid Interface Sci.

[24] Anzar N., Hasan R., Tyagi M., Yadav N., Narang J. (2020). Carbon nanotube - a review on synthesis, properties and plethora of applications in the field of biomedical science. Sens Int.

[25] Qureshi Z.A., Dabash H., Ponnamma D., Abbas M.K.G. (2024). Carbon dots as versatile nanomaterials in sensing and imaging: efficiency and beyond. Heliyon.

[26] Zhou Y., Liu K., Zhuang S., Mu Y. (2025). GO/CdS heterojunctions for accelerated photocatalytic antibiotic degradation. Nanomaterials.

[27] Shukla S., Khan R., Daverey A. (2021). Synthesis and characterization of magnetic nanoparticles, and their applications in wastewater treatment: a review. Environ Technol Innov.

[28] More P.R., Pandit S., Filippis A De, Franci G., Mijakovic I., Galdiero M. (2023). Silver nanoparticles: bactericidal and mechanistic approach against drug resistant pathogens. Microorganisms.

[29] Li H., Hua J., Li R. (2023). Application of magnetic nanocomposites in water treatment: Core–shell Fe3O4 material for efficient adsorption of Cr(VI). Water (Switzerland).

[30] El Messaoudi N., Aydın F., Miyah Y. (2025). Metal and metal oxide nanoparticles combined with ionic liquids and deep eutectic solvents and their practices in drug extraction and environmental processes: a review. J Mol Liq.

[31] Sirelkhatim A., Mahmud S., Seeni A. (2015). Review on zinc oxide nanoparticles: antibacterial activity and toxicity mechanism. Nano-Micro Lett.

[32] Liu M., Ye Y., Xu L., Gao T., Zhong A., Song Z. (2023). Recent advances in nanoscale zero-valent iron (nZVI)-Based advanced oxidation processes (AOPs): applications, mechanisms, and future prospects. Nanomaterials.

[33] Sharma P., Bhalla V., Dravid V. (2012). Enhancing electrochemical detection on graphene oxide-CNT nanostructured electrodes using magneto-nanobioprobes. Sci Rep.

[34] Aasli B., El Messaoudi N., Miyah Y. (2025). A comprehensive review of the use of urea-formaldehyde resin composites for the adsorption of organic and inorganic pollutants from wastewater. Nano-Struct Nano-Objects.

[35] Sutkar P.R., Gadewar R.D., Dhulap V.P. (2023). Recent trends in degradation of microplastics in the environment: a state-of-the-art review. J Hazard Mater Adv.

[36] Nguyen M.N., Jue M.L., Buchsbaum S.F. (2024). Interplay of the forces governing steroid hormone micropollutant adsorption in vertically-aligned carbon nanotube membrane nanopores. Nat Commun.

[37] Dianová L., Tirpák F., Halo M. (2022). Effects of selected metal nanoparticles (Ag, ZnO, TiO2) on the structure and function of reproductive organs. Toxics.

[38] Patel D.K., Kim H.B., Dutta S.D., Ganguly K., Lim K.T. (2020). Carbon nanotubes-based nanomaterials and their agricultural and biotechnological applications. Materials.

[39] Thakur A., Kumar A., Singh A. (2024). Adsorptive removal of heavy metals, dyes, and pharmaceuticals: Carbon-based nanomaterials in focus. Carbon.

[40] Miyah Y., Benjelloun M., Mejbar F. (2025). CWPO mechanism for toxic dye degradation in the presence of Cu@FbHAp catalyst: DFT study, performance analysis, response surface methodology, regeneration, and cost estimation. Results Chem.

[41] Aasli B., El Messaoudi N., El Mouden A. (2025). Synthesis of urea-formaldehyde resin@chitosan composite for the removal of Congo red from an aqueous solution via adsorption: Box-Behnken design optimization. Int J Biol Macromol.

[42] Latif M., Nawaz R., Aziz M.H. (2024). Evaluation of Tetracycline Photocatalytic Degradation Using NiFe2O4/CeO2/GO Nanocomposite for Environmental Remediation: In Silico Molecular Docking, Antibacterial Performance, Degradation Pathways, and DFT Calculations. Sep Purif Technol.

[43] Chen K., Dong W., Huang Y., Wang F., Zhou J.L., Li W. (2025). Photocatalysis for sustainable energy and environmental protection in construction: a review on surface engineering and emerging synthesis. J Environ Chem Eng.

[44] Cui L., Ren X., Sun M., Liu H., Xia L. (2021). Carbon dots: synthesis, properties and applications. Nanomaterials.

[45] Okpara E.C., Quadri T.W., Ebenso E.E., Rowley-Neale S.J., Banks C.E. (2025). Advancing water purification: the role of copper-carbon nanostructured heterojunctions in pollutant removal. Coord Chem Rev.

[46] Saroa A., Singh A., Jindal N. (2023). Nanotechnology-assisted treatment of pharmaceuticals contaminated water. Bioengineered.

[47] Badran I., Al‐Ejli M.O. (2022). Efficient multi‐walled carbon nanotubes/iron oxide nanocomposite for the removal of the drug ketoprofen for wastewater treatment applications. ChemistrySelect.

[48] Huaccallo-Aguilar Y., Álvarez-Torrellas S., Larriba M. (2021). Naproxen removal by CWPO with Fe3O4/multi-walled carbon nanotubes in a fixed-bed reactor. J Environ Chem Eng.

[49] Liang G., Hu Z., Wang Z., Yang X., Xie X., Zhao J. (2020). Effective removal of carbamazepine and diclofenac by CuO/Cu2O/Cu-biochar composite with different adsorption mechanisms. Environ Sci Pollut Control Ser.

[50] Duan C., Yu Y., Xiao J. (2020). Water-based routes for synthesis of metal-organic frameworks: a review. Sci China Mater.

[51] Hulloli S.K., Shivaraj B.W. (2015). Synthesis and characterization of Ag doped ZnO thin films. J Sol Gel Sci Technol.

[52] Xiao K., Xu Y., Cao X., Xu H., Li Y. (2022). 60 Years of the Loeb-Sourirajan Membrane: Principles, New Materials, Modelling, Characterization, and Applications.

[53] Sadat Z., Farrokhi-Hajiabad F., Lalebeigi F. (2022). A comprehensive review on the applications of carbon-based nanostructures in wound healing: from antibacterial aspects to cell growth stimulation. Biomater Sci.

[54] Preethi D.R.A., Philominal A. (2022). Green synthesis of pure and silver doped copper oxide nanoparticles using Moringa oleifera leaf extract. Mater Lett X.

[55] Khan S.A., Shahid S., Hanif S., Almoallim H.S., Alharbi S.A., Sellami H. (2021). Green synthesis of chromium oxide nanoparticles for antibacterial, antioxidant anticancer, and biocompatibility activities. Int J Mol Sci.

[56] Liang Q., Shao B., Tong S. (2021). Recent advances of melamine self-assembled graphitic carbon nitride-based materials: design, synthesis and application in energy and environment. Chem Eng J.

[57] Thole D., Balogun S.A., Modibane K.D., Mhlaba R., Botha E., Musyoka N.M. (2025). Preparation and characterization of metal oxide/carbon nanotube nanocomposites for photocatalytic and photo-electrocatalytic hydrogen production: a review. Int J Electrochem Sci.

[58] Dhila H., Bhapkar A., Bhame S. (2025). Metal oxide/biochar hybrid nanocomposites for adsorption and photocatalytic degradation of textile dye effluents: a review. Desalination Water Treat.

[59] Wang R., Lu K.Q., Tang Z.R., Xu Y.J. (2017). Recent progress in carbon quantum dots: synthesis, properties and applications in photocatalysis. J Mater Chem A Mater.

[60] Ahmadi S., Quimbayo J.M., Yaah V.B.K., de Oliveira S.B., Ojala S. (2025). A critical review on combining adsorption and photocatalysis in composite materials for pharmaceutical removal: pros and cons, scalability, TRL, and sustainability. Energy Nexus.

[61] Teo C.Y., Jong J.S.J., Chan Y.Q. (2022). Carbon-based materials as effective adsorbents for the removal of pharmaceutical compounds from aqueous solution. Adsorpt Sci Technol.

[62] Ponce S., Murillo H.A., Alexis F., Alvarez-Barreto J., Mora J.R. (2023). Green synthesis of nanoparticles mediated by deep eutectic solvents and their applications in water treatment. Sustainability (Switzerland).

[63] El Messaoudi N., Miyah Y., Al Qadr Imad, Wan-Mohtar W.A. (2024). Advancements in adsorption and photocatalytic degradation technologies of brilliant green from water: current status, challenges, and future prospects. Mater Today Chem.

[64] Chen H., Simoska O., Lim K. (2020). Fundamentals, applications, and future directions of bioelectrocatalysis. Chem Rev.

[65] El Messaoudi N., Miyah Y., Singh N. (2024). A critical review of Allura Red removal from water: advancements in adsorption and photocatalytic degradation technologies, and future perspectives. J Environ Chem Eng.

[66] Rubesh Ashok Kumar S., Vasvini Mary D., Suganya Josephine G.A., Riswan Ahamed M.A. (2024). Graphene/GO/rGO based nanocomposites: emerging energy and environmental application– review. Hybrid Adv.

[67] Ciğeroğlu Z., El Messaoudi N., Miyah Y. (2024). Recent advances in the removal of sunset Yellow dye from wastewater: a review. Sustain Mater Technol.

[68] Golmohammadi M., Hanafi-Bojd H., Shiva M. (2023). Photocatalytic degradation of ciprofloxacin antibiotic in water by biosynthesized silica supported silver nanoparticles. Ceram Int.

[69] Sun Y., Zhang W., Li Q., Liu H., Wang X. (2023). Preparations and applications of zinc oxide based photocatalytic materials. Adv Sensor Energy Mater.

[70] Louros V.L., Ferreira L.M., Silva V.G. (2021). Photodegradation of aquaculture antibiotics using carbon dots-tio2 nanocomposites. Toxics.

[71] Pawar O.Y., Li H., Patil O.A. (2025). Structural transformation of graphitic carbon nitride from 2D to 1D architectures for enhanced piezoelectric performance. Chem Eng J.

[72] Wang J., Wang S. (2022). A critical review on graphitic carbon nitride (g-C3N4)-based materials: preparation, modification and environmental application. Coord Chem Rev.

[73] Hui S. (2024). Recent advances and prospects of carbon nanotubes in nanomedicine: a mini review. Int J Nanosci.

[74] Shyamalagowri S., Bhavithra H.A., Akila N. (2024). Carbon-based adsorbents for the mitigation of polycyclic aromatic hydrocarbon: a review of recent research. Environ Geochem Health.

[75] Şenol Z.M., Arslanoğlu H., Keskin Z.S., Mehmeti V., El Messaoudi N. (2025). Biosorption of rhodamine B and sunset yellow dyes on cross-linked chitosan-alginate biocomposite beads: experimental and theoretical studies. Int J Biol Macromol.

[76] Sistanizadeh Aghdam M., Cheraghi M., Sobhanardakani S., Mohammadi A.A., Lorestani B. (2025). Facile fabrication of novel magnetic chitosan@Ag-MWCN nanocomposite for the adsorptive removal of ciprofloxacin from aqueous solutions. Sci Rep.

[77] Huaccallo-Aguilar Y., Álvarez-Torrellas S., Larriba M. (2019). Optimization parameters, kinetics and mechanism of naproxen removal by catalytic wet peroxide oxidation with a hybrid iron-based magnetic catalyst. Catalysts.

[78] Liu D., Li M., Li X., Ren F., Sun P., Zhou L. (2020). Core-shell Zn/Co MOFs derived Co3O4/CNTs as an efficient magnetic heterogeneous catalyst for persulfate activation and oxytetracycline degradation. Chem Eng J.

[79] Kong J., Li R., Wang F. (2018). Sulfate radical-induced transformation of trimethoprim with CuFe2O4/MWCNTs as a heterogeneous catalyst of peroxymonosulfate: mechanisms and reaction pathways. RSC Adv.

[80] Farzanegan Z., Tahmasbi M. (2023). Evaluating the applications and effectiveness of magnetic nanoparticle-based hyperthermia for cancer treatment: a systematic review. Appl Radiat Isot.

[81] Tian N., Giannakis S., Akbarzadeh L., Hasanvandian F., Dehghanifard E., Kakavandi B. (2023). Improved catalytic performance of ZnO via coupling with CoFe2O4 and carbon nanotubes: a new, photocatalysis-mediated peroxymonosulfate activation system, applied towards Cefixime degradation. J Environ Manag.

[82] Isari A.A., Mehregan M., Mehregan S., Hayati F., Rezaei Kalantary R., Kakavandi B. (2020). Sono-photocatalytic degradation of tetracycline and pharmaceutical wastewater using WO3/CNT heterojunction nanocomposite under US and visible light irradiations: a novel hybrid system. J Hazard Mater.

[83] Al-Musawi T.J., Rajiv P., Mengelizadeh N., Sadat Arghavan F., Balarak D. (2021). Photocatalytic efficiency of CuNiFe2O4 nanoparticles loaded on multi-walled carbon nanotubes as a novel photocatalyst for ampicillin degradation. J Mol Liq.

[84] Nawaz M., Shahzad A., Tahir K. (2020). Photo-Fenton reaction for the degradation of sulfamethoxazole using a multi-walled carbon nanotube-NiFe2O4 composite. Chem Eng J.

[85] Islam MdS., Kekre K.M., Shah T.A., Tsai P.C., Ponnusamy V.K., Andaluri G. (2025). Unraveling the complexities of microplastics and PFAS synergy to foster sustainable environmental remediation and ecosystem protection: a critical review with novel insights. J Hazard Mater Adv.

[86] Araújo E.S., Pereira M.F.G., da Silva G.M.G., Tavares G.F., Oliveira C.Y.B., Faia P.M. (2023). A review on the use of metal oxide-based nanocomposites for the remediation of organics-contaminated water via photocatalysis: fundamentals, bibliometric study and recent advances. Toxics.

[87] Al-Rawashdeh N.A.F., Allabadi O., Aljarrah M.T. (2020). Photocatalytic activity of graphene oxide/zinc oxide nanocomposites with embedded metal nanoparticles for the degradation of organic dyes. ACS Omega.

[88] Joseph A., Yelekar G.R., Vijayanandan A. (2024). Efficiency of titanium dioxide-reduced graphene oxide in carbamazepine removal: a comparative study of adsorption, photocatalysis, and ultrafiltration techniques. Appl Catal O: Open.

[89] Heu R., Ateia M., Yoshimura C., Awfa D., Punyapalakul P. (2020). Photocatalytic degradation of organic micropollutants in water by zr-mof/go composites. J Compos Sci.

[90] Bayode A.A., Vieira E.M., Moodley R. (2021). Tuning ZnO/GO p-n heterostructure with carbon interlayer supported on clay for visible-light catalysis: removal of steroid estrogens from water. Chem Eng J.

[91] Moharrami E., Keshipour S. (2025). Photocatalytic degradation of tetracycline antibiotic using nitrogen-doped reduced graphene oxide-supported titania/platinum nanoparticles. npj Mater Degrad.

[92] Zhang Z., Wang W., Liu F. (2025). Removal efficiency and mechanism of typical PPCPs onto novel cyclodextrin–graphene oxide composite adsorbent in aqueous solutions. Water (Switzerland).

[93] Wang J., Li P., Zhao Y., Zeng X. (2022). Nb/N Co-Doped layered perovskite Sr2TiO4: preparation and enhanced photocatalytic degradation tetracycline under visible light. Int J Mol Sci.

[94] Chi H., Cao P., Shi Q., Song C., Lv Y., Peng T. (2025). Photocatalytic degradation of Ciprofloxacin by GO/ZnO/Ag composite materials. Nanomaterials.

[95] Negarestani M., Mollahosseini A., Farimaniraad H., Ghiasinejad H., Shayesteh H., Kheradmand A. (2023). Efficient removal of non-steroidal anti-inflammatory ibuprofen by polypyrrole-functionalized magnetic zeolite from aqueous solution: kinetic, equilibrium, and thermodynamic studies. Separ Sci Technol.

[96] Utami M., Wang S., Fajarwati F.I., Salsabilla S.N., Dewi T.A., Fitri M. (2023). Enhanced photodegradation of rhodamine B using visible-light sensitive N-TiO2/rGO composite. Crystals (Basel).

[97] Kumari S., Singh V., Singh D. (2024). Nanoparticle synthesis advancements and their application in wastewater treatment: a comprehensive review. Curr Chem Lett.

[98] Fernández-Fernández V., Ramil M., Cela R., Rodríguez I. (2022). Solid-phase extraction and fractionation of multiclass pollutants from wastewater followed by liquid chromatography tandem-mass spectrometry analysis. Anal Bioanal Chem.

[99] de L Oliveira EG., de Oliveira H.P., Gomes A.S.L. (2020). Metal nanoparticles/carbon dots nanocomposites for SERS devices: trends and perspectives. SN Appl Sci.

[100] Jlassi K., Al Ejji M., Ahmed A.K. (2023). A carbon dot-based clay nanocomposite for efficient heavy metal removal. Nanoscale Adv.

[101] Nugroho D., Wannakan K., Nanan S., Benchawattananon R. (2024). The synthesis of carbon dots//zincoxide (CDs/ZnO-H400) by using hydrothermal methods for degradation of ofloxacin antibiotics and reactive red azo dye (RR141). Sci Rep.

[102] Muangmora R., Kemacheevakul P., Chuangchote S. (2023). Fiberglass cloth coated by coffee ground waste-derived carbon quantum dots/titanium dioxide composite for removal of caffeine and other pharmaceuticals from water. Heliyon.

[103] Silva V., Fernandes J.F.A., Tomás M.C. (2023). Enhanced solar driven photocatalytic removal of antibiotics from aquaculture effluents by TiO2/carbon quantum dot composites. Catal Today.

[104] Silva V., Invêncio I., Silva C.P., Otero M., Lima D.L.D. (2022). Photodegradation of oxolinic acid in aquaculture effluents under solar irradiation: is it possible to enhance efficiency by the use of TiO2/carbon quantum dots composites?. Chemosphere.

[105] Silva V., Louros V.L., Silva C.P. (2024). A solar flow photo-reactor for antibiotic removal from aquaculture effluents using TiO2/carbon quantum dots. Chemosphere.

[106] Bjelajac A., Florea I., Zamfir M., Nenez S.T., Cojocaru C.S. (2023). Photocatalytic active ZnO1-xSx@CNTs heteronanostructures. Nanotechnology.

[107] Mandal S.K., Paul S., Datta S., Jana D. (2021). Nitrogenated CQD decorated ZnO nanorods towards rapid photodegradation of rhodamine B: a combined experimental and theoretical approach. Appl Surf Sci.

[108] Alves D.M., Prata J.V., Silvestre A.J., Monteiro O.C. (2023). Novel C-dots/titanate nanotubular hybrid materials with enhanced optical and photocatalytic properties. J Alloys Compd.

[109] Santos G.O.S., Goulart L.L., Cordeiro-Junior P.M.P., Sánchez-Montes I., Lanza M.V.M. (2022). Pharmaceutical contaminants: ecotoxicological aspects and recent advances in oxidation technologies for their removal in aqueous matrices. J Environ Chem Eng.

[110] Goyal R., Sahu S., Mitra S. (2024). Nanocellulose-reinforced 4D printed hydrogels: thermoresponsive shape morphing and drug release. ACS Appl Polym Mater.

[111] Oldenkamp R., Hamers T., Wilkinson J., Slootweg J., Posthuma L. (2024). Regulatory risk assessment of pharmaceuticals in the environment: current practice and future priorities. Environ Toxicol Chem.

[112] Sharma S., Sharma M., Kumar R. (2024). Recent advances and mechanisms of microbial bioremediation of nickel from wastewater. Environ Sci Pollut Control Ser.

[113] Wang H., Wang Y., Dionysiou D.D. (2023). Advanced oxidation processes for removal of emerging contaminants in water. Water (Switzerland).

[114] Baaloudj O., Scrano L., Bufo S.A. (2025). Environmental fate, ecotoxicity, and remediation of heterocyclic pharmaceuticals as emerging contaminants: a review of long-term risks and impacts. Organics.

[115] Taoufik N., Boumya W., Janani F.Z., Elhalil A., Mahjoubi F.Z., Barka N. (2020). Removal of emerging pharmaceutical pollutants: a systematic mapping study review. J Environ Chem Eng.

[116] Alobaidi W., Nsaif A., Aljawhar N. (2024). Review article: biodegradation of pharmaceutical pollutants: challenges, mechanisms, and environmental implications. Basrah Res Sci.

[117] Ghosh P., Bairagi D., Hazra N., Jana S., Banerjee A. (2023). Carbon-dot-decorated silver and gold nanocomposites for antibacterial activity and degradation of organic dyes. ACS Appl Nano Mater.

[118] Zhang W., Xu H., Xie F. (2022). General synthesis of ultrafine metal oxide/reduced graphene oxide nanocomposites for ultrahigh-flux nanofiltration membrane. Nat Commun.

[119] Farhan A., Arshad J., Rashid E.U. (2023). Metal ferrites-based nanocomposites and nanohybrids for photocatalytic water treatment and electrocatalytic water splitting. Chemosphere.

